# Dysregulated Signaling at Postsynaptic Density: A Systematic Review and Translational Appraisal for the Pathophysiology, Clinics, and Antipsychotics’ Treatment of Schizophrenia

**DOI:** 10.3390/cells12040574

**Published:** 2023-02-10

**Authors:** Andrea de Bartolomeis, Licia Vellucci, Giuseppe De Simone, Benedetta Mazza, Annarita Barone, Mariateresa Ciccarelli

**Affiliations:** 1 Laboratory of Molecular Psychiatry and Translational Psychiatry, University School of Medicine of Naples “Federico II”, Via Pansini 5, 80131 Naples, Italy; 2 Unit of Treatment Resistant Psychosis, Section of Psychiatry, Department of Neuroscience, Reproductive Science and Odontostomatology, University School of Medicine of Naples “Federico II”, Via Pansini 5, 80131 Naples, Italy

**Keywords:** synaptic signaling, antipsychotics, postsynaptic density, treatment resistant schizophrenia, signalosome, connectome, synaptosome, synaptopathy

## Abstract

Emerging evidence from genomics, post-mortem, and preclinical studies point to a potential dysregulation of molecular signaling at postsynaptic density (PSD) in schizophrenia pathophysiology. The PSD that identifies the archetypal asymmetric synapse is a structure of approximately 300 nm in diameter, localized behind the neuronal membrane in the glutamatergic synapse, and constituted by more than 1000 proteins, including receptors, adaptors, kinases, and scaffold proteins. Furthermore, using FASS (fluorescence-activated synaptosome sorting) techniques, glutamatergic synaptosomes were isolated at around 70 nm, where the receptors anchored to the PSD proteins can diffuse laterally along the PSD and were stabilized by scaffold proteins in nanodomains of 50–80 nm at a distance of 20–40 nm creating “nanocolumns” within the synaptic button. In this context, PSD was envisioned as a multimodal hub integrating multiple signaling-related intracellular functions. Dysfunctions of glutamate signaling have been postulated in schizophrenia, starting from the glutamate receptor’s interaction with scaffolding proteins involved in the N-methyl-D-aspartate receptor (NMDAR). Despite the emerging role of PSD proteins in behavioral disorders, there is currently no systematic review that integrates preclinical and clinical findings addressing dysregulated PSD signaling and translational implications for antipsychotic treatment in the aberrant postsynaptic function context. Here we reviewed a critical appraisal of the role of dysregulated PSD proteins signaling in the pathophysiology of schizophrenia, discussing how antipsychotics may affect PSD structures and synaptic plasticity in brain regions relevant to psychosis.

## 1. Introduction: The PSD Landscape, Receptors, Adaptors, and Scaffolding Proteins Linking to Schizophrenia

Among the major components of the glutamatergic synaptic complex, the postsynaptic density (PSD) is a finely specialized structure that lies beneath the postsynaptic membrane [1]. Several lines of evidence converge in envisioning PSD as a molecular switchboard that dispatches with spatial and temporal differentiation multimodal intracellular signaling from receptors lying on the postsynaptic surface, including N-methyl-D-aspartate receptor (NMDAR), metabotropic glutamate receptor (mGluR) type I, α-amino-3-hydroxy-5-methyl-4-isoxazole propionate receptor (AMPAR) kainate receptor, to PSD proteins acting as effectors/scaffolds, including postsynaptic density protein 95 (PSD-95), disrupted in schizophrenia 1 (DISC1), stargazin, Homer, SH3, and multiple ankyrin repeat domains (Shank), and finally to vesicular compartments with both Ca^2+^-dependent and Ca^2+^-independent mechanism [1]. Of interest, PSD proteins have been implicated in modulating behaviors in different species, such as invertebrates, mammals, primates, and humans [2,3,4,5]. Specifically, among the scaffold proteins, Homer seems to be responsible for the regulation of the wake–sleep cycle in drosophila melanogaster, as well as in mice and humans [6]. Moreover, gene-wide association studies (GWAS) have indicated PSD proteins’ genes are among the ones highly associated with schizophrenia [7]. Based on this evidence, it was hypothesized that the PSD structure, in addition to “reacting” to direct activation of hypofunctioning glutamate receptors, could potentially be involved in a more complex interaction of multiple altered intracellular mechanisms involving postsynaptic scaffold proteins resulting in an altered cortical-subcortical dopamine-glutamate interplay [8,9]. Despite the amount of PSD proteins (more than 1000), in the present study, we have focused on those resulting from the research conducted and reported in the methods section. Furthermore, clinical and preclinical evidence underlines the relevance of these PSD proteins and their translational implications for antipsychotic treatment in schizophrenia [10,11,12]. Therefore, we performed a gene set enrichment analysis (GSEA) to test whether the 12 different gene classes identified were over-represented in a large set of genes that might have an association with disease phenotypes using the Enrichr tool [13,14,15]. The analysis resulted that the gene set extracted by the systematic review is strongly associated with genes differentially expressed in schizophrenia (*p* ≤ 0.0001422) with reference to *DISC1* and *NRG1* [16]. Furthermore, considering that PSD genes are part of the genetic framework of schizophrenia vulnerability, some authors have linked biomarkers and complex disease-associated loci to a set of genes encoding human druggable proteins via GWAS, suggesting that genes encoding for PSD are a promising target for new treatments in schizophrenia [7].

This systematic review attempts to shed some light on the basic aspects concerning the role of PSD in schizophrenia pathophysiology, tackling the following questions:

1. How is PSD involvement coherent within the present framework of aberrant dopamine-glutamate interaction in schizophrenia?
2. What is the contribution of each PSD protein associated with the pathophysiology of schizophrenia?
3. How might innovative methodology and discovery contribute to a non-canonical conceptualization of PSD’s role in schizophrenia?
4. Finally, how does antipsychotic treatment impact PSD proteins, and how might it be instrumental in developing new therapeutic strategies for schizophrenia?

## 2. Materials and Methods

**Figure 1:**
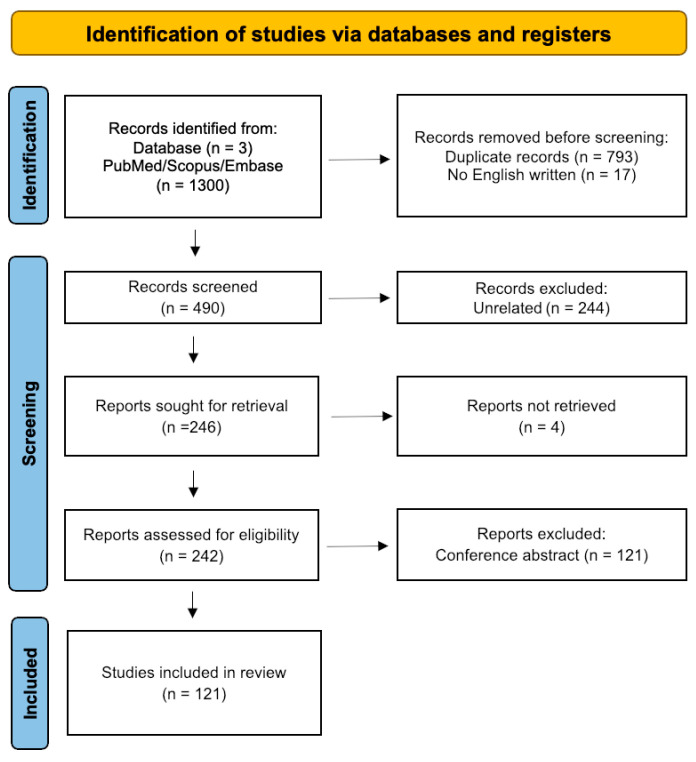
PRISMA flow diagram showing the flow of information through the different phases of the systematic review.

The present systematic review aims to provide an updated overview of the available evidence on the implication of PSD proteins in dysregulated signaling transduction and their potential implications in antipsychotic treatment in schizophrenia. The search and selection process were conducted according to the preferred reporting items for systematic reviews and meta-analyses (PRISMA) guidelines [17] in order to identify eligible clinical and preclinical studies investigating the signaling dysregulations at the PSD in schizophrenia. Therefore, the following searches were conducted in EMBASE, Scopus, and PubMed on 1 August 2022 (the last interrogation was conducted in 28 October 2022): (psd) AND (schizophrenia) + “psd schizophrenia” OR (psd AND (“schizophrenia”/exp OR schizophrenia)); ((schizophrenia) AND (psd)) AND (antipsychotic*) + (“schizophrenia”/exp OR schizophrenia) AND psd AND “antipsychotic*”; ((schizophrenia) AND (psd)) AND (clozapine) + (“schizophrenia”/exp OR schizophrenia) AND psd AND “clozapine”; ((schizophrenia) AND (psd)) AND (aripiprazole) + (“schizophrenia”/exp OR schizophrenia) AND psd AND “aripiprazole”; ((schizophrenia) AND (psd)) AND (brexpiprazole) + (“schizophrenia”/exp OR schizophrenia) AND psd AND “brexpiprazole”; ((schizophrenia) AND (psd)) AND (cariprazine) + (“schizophrenia”/exp OR schizophrenia) AND psd AND “cariprazine”; ((schizophrenia) AND (psd)) AND (risperidone) + (“schizophrenia”/exp OR schizophrenia) AND psd AND “risperidone”; ((schizophrenia) AND (psd)) AND (haloperidol) + (“schizophrenia”/exp OR schizophrenia) AND psd AND “haloperidol”; ((schizophrenia) AND (psd)) AND (olanzapine) + (“schizophrenia”/exp OR schizophrenia) AND psd AND “olanzapine”; ((schizophrenia) AND (psd)) AND (chlorpromazine) + (“schizophrenia”/exp OR schizophrenia) AND psd AND “chlorpromazine”; ((schizophrenia) AND (psd)) AND (fluphenazine) + (“schizophrenia”/exp OR schizophrenia) AND psd AND “fluphenazine”; ((schizophrenia) AND (psd)) AND (lurasidone) + (“schizophrenia”/exp OR schizophrenia) AND psd AND “lurasidone”; ((schizophrenia) AND (psd)) AND (xanomeline) + (“schizophrenia”/exp OR schizophrenia) AND psd AND “xanomeline”; ((schizophrenia) AND (psd)) AND (quetiapine) + (“schizophrenia”/exp OR schizophrenia) AND psd AND “quetiapine”; ((schizophrenia) AND (psd)) AND (iloperidone) + (“schizophrenia”/exp OR schizophrenia) AND psd AND “iloperidone”; ((schizophrenia) AND (psd)) AND (sulpiride) + (“schizophrenia”/exp OR schizophrenia) AND psd AND “sulpiride”; ((schizophrenia) AND (psd)) AND (amisulpride) + (“schizophrenia”/exp OR schizophrenia) AND psd AND “amisulpride”; ((schizophrenia) AND (psd)) AND (asenapine) + (“schizophrenia”/exp OR schizophrenia) AND psd AND “asenapine”; ((schizophrenia) AND (psd)) AND (bromperidol) + (“schizophrenia”/exp OR schizophrenia) AND psd AND “bromperidol”; ((schizophrenia) AND (psd)) AND (clotiapine) + (“schizophrenia”/exp OR schizophrenia) AND psd AND “clotiapine”; ((schizophrenia) AND (psd)) AND (loxapine) + (“schizophrenia”/exp OR schizophrenia) AND psd AND “loxapine”; ((schizophrenia) AND (psd)) AND (paliperidone) + (“schizophrenia”/exp OR schizophrenia) AND psd AND “paliperidone”; ((schizophrenia) AND (psd)) AND (perphenazine) + (“schizophrenia”/exp OR schizophrenia) AND psd AND “perphenazine”; ((schizophrenia) AND (psd)) AND (pimozide) + (“schizophrenia”/exp OR schizophrenia) AND psd AND “pimozide”; ((schizophrenia) AND (psd)) AND (promazine) + (“schizophrenia”/exp OR schizophrenia) AND psd AND “promazine”; ((schizophrenia) AND (psd)) AND (thioridazine) + (“schizophrenia”/exp OR schizophrenia) AND psd AND “thioridazine”; ((schizophrenia) AND (psd)) AND (tiapride) + (“schizophrenia”/exp OR schizophrenia) AND psd AND “tiapride”; ((schizophrenia) AND (psd)) AND (trifluoperazine) + (“schizophrenia”/exp OR schizophrenia) AND psd AND “trifluoperazine”; ((schizophrenia) AND (psd)) AND (ziprasidone) + (“schizophrenia”/exp OR schizophrenia) AND psd AND “ziprasidone”. The PRISMA flow diagram is reported in Figure 1. We deemed eligible English-written articles, published in peer-reviewed journals, exploring the involvement of dysregulated signaling of PSD proteins in schizophrenia and their implication in antipsychotic treatment. No time constraints were applied, and original clinical and preclinical research studies and reviews were included. Conference abstracts and commentaries were excluded. The search returned a total of 1300 articles. One hundred and twenty-one articles were included in the qualitative synthesis and divided into topics of interest. The selection of articles from the primary search was conducted independently by two authors, and, in case of inconsistent evaluation, a third author’s opinion was requested. Furthermore, in addition to the primary PRISMA search and after having evaluated the abstract and full text of each article of the final selection, a manual and ad hoc search was performed with a focus on PSD proteins with the highest citation for putative association/involvement in schizophrenia. This additional search was necessary when the primary mechanisms or functions of PSD proteins needed to be precise in light of the other molecular mechanisms of schizophrenia pathophysiology (i.e., dopamine–glutamate interaction). Although this may introduce a search bias, we should acknowledge it; nevertheless, we believe that for the present topic, mainly a translational review linking preclinical and clinical data, which is different from a more oriented clinical systematic review (i.e., interventional or observational ones), the overall strategy has allowed us to deepen the mechanistic aspects of PSD involvement in schizophrenia. We registered the review protocol on the International Platform of Registered Systematic Review and Meta-analysis Protocols (INPLASY2022110129). In addition, after reading the entire text of the selected article, other cross-references were included.

## 3. The PSD Definition and Structure

### 3.1. The Spatial and Temporal Distribution of PSD

Recent evidence has highlighted the relevance of PSD proteins, such as PSD-95, Homer, Shank, and DISC1, in the pathophysiology of major psychiatric disorders, including schizophrenia and autism spectrum disorder (ASD). A highly conserved set of ~1000 PSD proteins is shared among vertebrates, and differences in the proteome indicate how species have evolved distinct synapse types and functions [18]. The PSD is a thickness of approximately 300–500 Angstrom length detectable with electron microscopy at the postsynaptic membrane of glutamatergic synapses. Applying two different types of targeted mass spectrometry-based assays in mouse cortical brain tissue, 50 PSD proteins have been detected with a parallel reaction monitoring (PRM) assay using heavy-labeled, synthetic internal peptide standards, whereas 2100 proteins were identified based on a pre-determined spectral library in PSD fractions [19]. Two major layers have been identified with electron microscopy in the overall structure of the PSD that is from the external part of the membrane surface are: (1) the “core” immediately beneath the neuronal membrane and (2) the “pallium” based on the typology of proteins therein localized and composed of scaffold proteins, receptors, adaptors, and inhibitory proteins. It is considered a structural and functional intersection where several neurotransmitter systems, such as glutamatergic, dopaminergic, and serotoninergic, converge and is believed to have a potentially relevant implication in the pathophysiology of psychosis [20]. With regard to glutamatergic neurotransmission, NMDAR, which is considered the core of the structure in concert with AMPAR and mGluR type I (mGluR 1 and mGluR 5) [21] plays a crucial role in the PSD complex. The most abundant membrane-associated guanylate kinase family (MAGUK) of mammalian PSD is postsynaptic density protein 95 (PSD-95), discs large 4 (DLG4), and synapse-associated protein 90 (SAP90). Others include SAP97 (DLG1), PSD-93 (DLG2, Chapsyn-110), SAP102 (DLG3), guanylate kinase-associated protein (GKAP), also called SAPAP (SAP90/PSD-95-associated protein), SH3 and multiple ankyrin repeat domains protein (Shank) also known as ProSAP (proline-rich synapse-associated protein), Homer otherwise called Vesl, Cupidin, and PSD-Zip45. Thus, in the dense matrix of PSD, the most represented scaffolding and adaptor proteins associated with schizophrenia pathophysiology, are PSD-95, Homer, and Shank. These interacting proteins, which have the role of linking membrane surface receptors through their structure and function, modulate the intracellular signaling mechanism [22]. The PSD has emerged in the last few years as a relevant synaptic element involved in shaping the dendritic spines and regulating multiple cell transduction signaling, whose dysregulation has been linked to synaptic aberration in neurodevelopmental disorders [11,23,24]. In addition, multiple lines of evidence suggest that antipsychotic treatments, including novel interventions in refractory psychosis [12], impact PSD gene expression [9]. Electron microscopy studies identified a three-dimensional laminate network of scaffolding proteins that organizes signal detection and modulation by transducing a postsynaptic signal. This architecture is derived from the binding properties and characteristics of each protein of PSD [25]. PSD-95 is located in a layer just below the postsynaptic membrane, in a position favoring interaction between glutamate receptors, ion channels, signaling proteins, and cell adhesion molecules (CAMs) and clusters them on the postsynaptic membrane. Scaffold molecules not anchored to the postsynaptic membrane, such as Shank, guanylate-kinase associated proteins (GKAP), and Homer, fill a deeper layer within the PSD and favor the connection of synaptic membrane surface to cytoskeletal components (such as α-actinin, cortactin, and actin), regulating the size and shape of dendritic spines [26]. Centrally placed within the PSD lies the Shank family of proteins and their binding partners that form large multi-protein complexes and are considered the main scaffold within the PSD cytomatrix [27]. Moreover, PSD mainly consists of cytoskeletal proteins and calcium/calmodulin-dependent protein kinase II (CaMKII), while traditional scaffold proteins, such as PSD-95 and Shank1, are less represented components [22]. The temporal distribution of synaptic proteins is considered crucial for starting, modulating, and ending the intracellular signaling originating at the receptor level and conducted by adaptors, scaffolding, and effector proteins. In this regard, recently, coupling the halo-binding site/ligand technology with synaptosome mapping pipeline technology (SYNMAP) has allowed linking the HaloTag ligand to fluorophores, enabling irreversible labeling and visualization of tagged proteins [28]. These approaches could deepen the interaction of multiple PSD proteins, their spatial and temporal distribution in dendritic spines, and their mutual interplay.

### 3.2. Glutamate Receptors: NMDAR and Beyond

At the level of excitatory synapses, it has been shown that approximately 2000–3000 glutamate molecules are released from the synaptic vesicle, whereas only ∼50–100 ionotropic glutamate receptors are expressed by a single PSD. Therefore, more than 90% of glutamate molecules will escape the synaptic cleft even when the maximal binding capacity (B_max_) of the ionotropic glutamate is elevated, as the B_max_ is the total number of receptors detectable in brain tissue by a binding assay of reference radioligands, for example [^3^H]MK801 for the NMDAR, (S)-[^3^H]-5-fluorowillardiine for the AMPAR, or [^3^H]kainic for kainite [29]. Furthermore, it has been shown that within a millisecond, the concentration of glutamate at the release site decreases by an order of magnitude. In this highly dynamic scenario, it is conceivable that the concentration, localization, diffusion, and activation/inactivation of the receptors must be precisely regulated, and PSD proteins are crucial to this mechanism [30,31,32,33]. Several types of glutamate receptors are grouped within the PSD, including metabotropic glutamate receptors, which mediate transmembrane signal transduction through interactions with G-proteins, along with ionotropic glutamate receptors, such as NMDAR, AMPAR, and kainate receptors. Among these, the two principal classes, NMDAR and AMPAR, are highly enriched in the PSD and are crucial for translating glutamate into electrical depolarization at the postsynaptic level. The binding of these receptors to PSD is mediated by physical interaction with a large number of supporting molecules, including PSD-95, guanylate kinase-associated protein (GKAP), glutamate receptor-interacting protein (GRIP), proteins interacting with C kinase-1 (PICK1), Shank, and Homer. Preclinical studies showed that the long-term potentiation (LTP) induction threshold decreased with a down-regulation of NMDAR in PSD-95 knockout (KO) mice, modulating the synaptic plasticity through the PSD-95 multi-protein complex [34]. These structural proteins form a PSD lattice-based dynamic structure, stabilizing membrane proteins and matching glutamate receptors to presynaptic release sites [22]. Scaffolding proteins have several common (PDZ)-binding domains, including discs large (Dlg) and zonular occludens-1 (ZO-1), which support the tethering of many other membrane-associated and scaffolding molecules. The PDZ domains allow binding to a plethora of signaling molecules, such as CAMKII or the GTPases Ras, Rho, Rac, and Arf, making possible the various signaling cascades downstream of glutamate receptors [35]. Further molecules that play a pivotal role in the spatial organization of the PSD and in keeping the synapse together are cytoskeletal proteins, such as actin, tubulin, and cortactin, which interact with numerous molecules of this complex scaffold in a highly dynamic mode [36,37].

## 4. The Shank Family and Schizophrenia: Overlapping with Other Neurodevelopmental Diseases

### 4.1. Shank Structure and Topography

The ProSAP/Shank family is constituted of PSD scaffolding proteins that are implicated in propagating and modulating glutamate neurotransmission [38]. Shank genes derive from the same orthologous genes, with slight differences in humans and animals, thus providing an optimal candidate for translational research in neuropsychiatric disorders [39]. Shank proteins (i.e., Shank1, Shank2, and Shank3) are coded by three different genes, each of them having multiple splice variants producing different Shank proteins [40]. The distinct Shank isoforms differ in their expression pattern. Shank1 is expressed in the rat brain [40,41] with high abundance in the cortex, hippocampus, and amygdala, with lower expression in the thalamus and substantia nigra and negligible levels in the cerebellum, caudate nucleus, corpus callosum and subthalamic nucleus [41]. Shank2 is strongly expressed in the cortex, hippocampus, Purkinje cells, olfactory bulb, and central grey cells [42]; Shank3, finally, is limitedly expressed in the rat brain [40]. At the PSD, these proteins are capable of interacting with structural elements as well as ligand-gated ion channels, cell adhesion molecules, such as neuroligins, and proteins regulating the dynamic assembly of filamentous actin (F-actin) [43]. Shank3-dependent molecular, structural, and functional changes of excitatory synapses have been characterized from preclinical in vivo studies in a complete KO mouse model of the autism-associated *Shank3* gene, underlying pathophysiological mechanisms [44]. This model demonstrated that Shank3 deficiency might impair the mGluR5-Homer interaction, resulting in cortico-striatal circuit abnormalities that underlie learning deficits and ASD-like behavior, providing a novel potential therapeutic approach for Shank3-associated functional and behavioral defects in neuropsychiatric disorders [44,45].

### 4.2. Shank Connectome and Signalosome 

Shank proteins may cross-link Homer and PSD-95 at the PSD and participate in NMDAR, mGluR, and AMPAR downstream signaling [46]. Moreover, Shank proteins promote spine formation, as well as maturation and enlargement of dendritic spines [47]. Through their multiple interactions, Shank proteins enable the formation of a polymeric network complex, which requires the assembly of Homer tetramers and Shank multimers, forming a structure that has been proposed to serve as a functional platform for other PSD proteins [38]. Multiple lines of evidence showed that ASD-associated mutations in Shank3 may impair AMPAR and NMDAR signaling and may alter neurexin–neuroligin-mediated signaling in cultures of rat hippocampal neurons [48]. These findings may suggest that Shank protein aberrations could contribute to cognitive symptoms in intellectual disability and could also be implicated in schizophrenia [48]. Shank abnormalities have been described to impair glutamate-mediated neurotransmission and the morphology of dendritic spines. Indeed, Shank proteins appear to be critically involved in regulating the morphology, architecture, and function of dendritic spines and in modulating glutamatergic signaling relevant to several neurodevelopmental disorders [49], such as schizophrenia [50]. Taken together, this evidence demonstrates a critical role for Shank in the normal development of neuronal connectivity and establishes causality between a disruption in the *Shank* genes and the biological basis of neuropsychiatric disorders [51].

### 4.3. Shank and Schizophrenia

Dysfunctions of Shank proteins have been demonstrated in several neuropsychiatric disorders, i.e., ASD, schizophrenia, and Alzheimer’s disease [52]. Schizophrenia and ASD are characterized by overlapping symptoms, particularly negative symptoms, suggesting that they share a common biological basis in their pathogenesis [53]. These observations are reflected in the rare risk mutations of the *Shank* gene family that are increased in patients with schizophrenia and ASD compared with healthy controls (HC), underlying some of the cognitive and social dysfunction relevant for both these conditions [10]. The involvement in ASD and intellectual disability of Shank has been repeatedly demonstrate. Specifically, deletions of the *Shank1* gene have been described in altered social behavior in *Shank1*^−/−^ mutant mice, including decreased levels of ultrasonic vocalizations and scent-marking behavior [54]. Several pieces of evidence on the role of *Shank* mutations have suggested alterations of the dendrite number and spiny head enlargement in Shank1 mutants [55]. On the other hand, *Shank2* and *Shank3* mutants showed a significant reduction in the dendritic spine of cerebellar Purkinje cells and the striatal spines density, respectively [56,57], suggesting that these isoforms might have a higher impact on the induction and maturation of dendritic spines [55]. *Shank2* mutant mice showed a decreased number of dendritic spines, basal synaptic transmission, miniature frequency [58], and NMDAR function, normalized by administration of an NMDAR partial agonist with improved behavioral abnormalities and social interaction [59]. Mutations in the gene encoding for Shank3 have been reported in ASD patients [60], and a variety of point mutations and small deletions of *Shank3* have been causally associated with numerous neurodevelopmental and neuropsychiatric disorders, including schizophrenia [61,62] (Table 1). Of interest, mice carrying *Shank3* deletions exhibit self-injurious, repetitive grooming behaviors and deficits in social interaction reminiscent of autistic behaviors in humans [63]. Notably, de novo mutations of *Shank3* have been reported in cases of schizophrenia and schizoaffective disorder; specifically, the *R1117X* mutation can cause loss of function of the Shank3 protein [64]. Moreover, duplications of *Shank3* have also been reported in patients with bipolar disorder [65] and schizophrenia [66]. The transgenic mice that mildly overexpress Shank3 proteins display manic-like hyperkinetic behaviors [67], potentially implicated in multiple neuropsychiatric disorders [68]. These results suggest that the proper expression and function of Shank3 are critical for normal synaptic development and function. Proteomic and genomic evidence involving PSD-associated proteins and genes implicated in schizophrenia showed altered differentially expressed NMDA-interacting proteins, such as Shank3, known to contribute to the pathophysiology of the disease [69].

### 4.4. Shank and Psychotropic Drugs

Several studies have shown that antipsychotic treatments can affect the formation of the dendritic spine and synaptogenesis by direct rearrangement of the synaptic structure, regulating postsynaptic scaffolding proteins, such as Shank (Table 2). Atypical and typical antipsychotics are shown to differentially modulate the protein density at dendritic spines. Specifically, an in vitro study on rat hippocampal neurons demonstrated that clozapine administration increased Shank1a protein density along dendrites, whereas Shank1a protein density was decreased by haloperidol [102]. The differential modulation of *Shank* expression by clozapine and haloperidol may be consistent with a different impact on dendritic spine formation, suggesting the possible contribution of clozapine to higher efficacy in severe conditions, such as treatment-resistant schizophrenia [103]. Some authors have pointed out that chronic treatment with mood stabilizers, lithium, or valproate, used clinically as antipsychotic augmentation strategies in schizophrenia, can directly affect the expression of Homer1b/c and related Shank and inositol 1,4,5 trisphosphate receptors (IP3Rs) in cortical and subcortical regions [20,104]. In particular, Shank expression was affected by lithium in the cortex, whereas valproate was shown to downregulate gene expression in the motor and insular cortex [104]. However, lithium treatment significantly reduced IP3R in almost all cortical subregions, whereas valproate reduced it in the dorsolateral caudate-putamen [104]. Shank proteins interact with NMDARs through a PSD-95/GKAP complex and with mGluR1/5 by involving Homer1b/c, which via IP3Rs bind this machinery to intracellular Ca^2+^ release [39]. Specifically, Homer1b/c, Shank, and the IP3Rs play an essential role regulating intracellular Ca^2+^ oscillations with a potential neuroanatomical specificity, suggesting a differential intracellular Ca^2+^ networks regulation by mood stabilizers [46,105]. Therefore, the impact of mood stabilizers’ chronic administration on Shank and IP3R transcripts in cortical and subcortical regions might regulate calcium-dependent glutamatergic activity. Furthermore, the fact that Shank was modulated in brain regions specifically involved in cognitive, behavioral, and motor functions may further confirm its essential role in transductive pathways implicated in the mechanism of action of psychotropic drugs [104].

## 5. The PSD-95

### 5.1. PSD-95 Structure and Topography

PSD-95, also known as synapse-associated protein 90 (SAP-90), is the most abundant protein of the PSD [151,152], belonging to the membrane-associated guanylate kinase family (MAGUK). It is a scaffolding protein located at excitatory synapses and involved in the glutamatergic neurotransmission and trafficking of NMDARs and AMPARs [153,154]. PSD-95 presents three N-terminal PDZ domains (PSD-95, Dlg, and ZO-1), an src homology domain (SH3), and a guanylate cyclase domain (GUK) [152,155]. Through the PDZ domain, PSD-95 can directly connect with the C-terminus of NR2A and NR2B subunits of NMDAR and to AMPAR via stargazin/TARPs [156] while an indirect interaction is established with mGluR1/5 via GKAP [157,158,159,160]. Based on its involvement as a key component of PSD development and function, PSD-95 levels increase during development with maximum levels in adults, consistent with synaptic density growth in the human PFC [161]. Furthermore, PSD-95 localization is defined also through its post-translational modifications, such as palmitoylation [162,163] at Cys3 and Cys5 obtained by continuous palmitoylation/depalmitoylation cycles, necessary for its interaction with K+ channel KV1.4 and its clustering at the PSD [162,164,165].

### 5.2. PSD-95 Connectome and Signalosome

PSD-95 is an essential protein in the synaptic plasticity and dendritic spine morphogenesis during the neurodevelopment process, acting by the stabilization, recruitment, and trafficking of glutamatergic receptors [155,166,167]. Specifically, the PSD-95 effect on NMDAR localization and stabilization at the PSD is different depending on the period of life. In fact, it has been demonstrated in animal models that the expression levels of PSD-95 increase from early life to adulthood along with an elevation in NR2A-subunit levels and differently from SAP102, showing an opposite trend [161,168,169] (Table 1). Thus, during neurodevelopment until adult age, a critical switch from the SAP102/NR2B-NMDAR to PSD-95/NR2A-NMDAR complexes has highlighted the nodal role of PSD-95 as a mediator of NMDAR, consistent to prevent the hyperexcitability at the synapses [170,171]. The relevance of PSD-95 in the neurodevelopmental process is confirmed also by a recent study in which the reduced levels of the protein affect NMDAR and AMPAR function and levels in the medial prefrontal cortex (mPFC) during juvenile and adolescent periods. This study showed an increase in NR1 and NR2A, and a decrease in GluA1 subunit with behavioral alterations including lack of sociability, learning, and working memory deficits [171]. Concerning glutamatergic neurotransmission, PSD-95 is found to form a quinary complex including AMPAR, stargazin, serine racemase (SR), and the NMDAR [92]. SR binds to the third PDZ domain of PSD-95, while stargazin binds to the first and second PDZ domains [92]. AMPAR activation is responsible for the dissociation of SR from the complex and an increase in catalytic activity, favoring NMDAR transmission mediated by D-serine elevation [92]. The regulation of NMDAR by PSD-95 seems to be related also to a cytosolic sulfotransferase 4A1 involved in several neuropsychiatric disorders [172,173,174] that restore the correct processes of synaptic plasticity and the dendritic spine density [85,175,176]. PSD-95 also forms a complex with synaptosomal-associated protein 25kDa (SNAP-25) and p140Cap, opening the possibility that reduction in its levels may contribute to affect postsynaptic function and plasticity as demonstrated in SNAP-25 heterozygous mice with reduced densities of dendritic spines [87]. A decrease in PSD-95 levels is also reported in conditional heparin-binding EGF-like growth factor KO mice that are found altered in the brain and serum of patients affected by schizophrenia [177], underlining its crucial role in the pathophysiology of the disease [95]. PSD-95 is also involved in the regulation of inhibitory neurotransmission. In fact, transgenic adolescent mice models (PSD-95^+/−^) have shown a significant increase in the α1 subunit of GABA_A_ in medial PFC with subsequent increases in the frequency and amplitude of spontaneous inhibitory postsynaptic currents (sIPSCs) in pyramidal neurons, responsible for a shift from excitatory to inhibitory balance [94]. In a preclinical study, Abbas et al. demonstrated that 5-hydroxy-tryptamine-(5HT)_2A_-and 5-HT_2C_-mediated downstream signaling were impaired in PSD-95^null^ mice, suggesting the role of PSD-95 in modulating and scaffolding macromolecular glutamatergic signaling complexes and metabotropic 5HT_2A_ and 5HT_2C_ receptors function [178]. PSD-95 is also directly connected to D1R and forms a ternary complex with Calcyon that regulates the entry of Ca^2+^ by the membrane, playing a role in the internalization of D1R through its phosphorylation [179].

### 5.3. PSD-95 in Schizophrenia and Relevance for Antipsychotic Treatment

Several human and animal studies suggest that PSD-95 disruption is involved in the neuropathology of schizophrenia and ASD [180,181]. A recent preclinical study using mice with loss-of-function mutations in the PSD-95 protein complex and an innovative cognitive touchscreen test showed that distinct components differentially regulate learning, cognitive flexibility, and reaction times in cognitive processing. This study provided new insights into how mutations in this molecular complex could lead to different and complex phenotypes in neurodevelopmental disorders [182]. In addition, post-mortem studies have reported a reduction in PSD-95 mRNA and proteins in the dorsolateral prefrontal cortex (DLPFC) and dorsomedial prefrontal cortex of patients affected by schizophrenia compared with HC [126,183] (Table 2). A decrease in PSD-95 protein levels was found also in the *cornu ammonis 1* of the hippocampus, a region that is critically involved in schizophrenia patients in comparison with matched controls [111]. These data are also confirmed by another post-mortem study where the PSD-95 expression was assessed in the frontal cortex, using immunoprecipitation and Western blotting in schizophrenia compared with HC, adding to existing evidence for disturbed postsynaptic glutamate function and synaptic plasticity in schizophrenia [134]. Furthermore, in preclinical studies conducted on mice, deletion in the gene Discs Large MAGUK Scaffold Protein 4 (DLG4) encoding for PSD-95, is associated with behavioral and molecular abnormalities, consisting of the clinical phenotype of repetitive behavior and irregular social interactions [184]. In addition, an immunoprecipitation study with Western Blot analyzed the scaffolding protein DLGAP1 localized in glutamatergic neurons, which is a component of protein complexes organized by PSD95. In this context, *Dlgap1* KO mice exhibited disruption of protein interactions in PSD and sociability deficits, consistent with the findings of DLGAP1 haploinsufficient variants in schizophrenia and ASD [83]. Given the crucial role of PSD-95 in the structure and architecture of PSD, as well as in its signaling and function, several lines of evidence have demonstrated modulation by antipsychotics or psychotropic compounds that targeted these molecules [107,114]. Multiple preclinical studies have demonstrated their impact on PSD-95 mRNA expression. It has been demonstrated that an increase in dorsolateral and ventromedial caudate-putamen (*p* = 0.015 and *p* = 0.04, respectively) after memantine administration compared with MK-801, supporting the view that memantine differently modulates key PSD molecules contributing to the different activities on neuroplasticity exerted by this compound [114]. On the other hand, the administration of antipsychotics is involved in the modulation of PSD-95, in particular, asenapine, at doses of 0.05 mg/kg, 0.1 mg/kg, and 0.3 mg/kg, is responsible for increased mRNA levels in all cortical and subcortical regions assessed: anterior cingulate cortex (ACC), medial agranular cortex, motor cortex, somatosensitive cortex, insular cortex, dorsomedial caudate-putamen, dorsolateral caudate-putamen, ventrolateral caudate-putamen, ventromedial caudate-putamen, NAc, nucleus accumbens shell. Haloperidol at 0.5 mg/Kg is found to induce overexpression of PSD-95 in the ACC and motor cortex, while at the dose of 0.8 mg/Kg it is responsible for its increase in the ACC, motor cortex, somatosensory cortex, insular cortex, as well as in the caudate-putamen dorsolateral, dorsomedial, ventromedial, NAc, and nucleus accumbens shell [107]. Additionally, chronic administration of antipsychotics haloperidol and ziprasidone were evaluated at 90 min (early time-point) or 24 h (delayed time-point), where PSD-95 expression was found to be significantly increased in ventromedial caudate-putamen at the delayed time-point by ziprasidone compared with vehicle and by haloperidol in the striatum at the early time-point compared with delayed time-point [119]. In this context, ziprasidone increased PSD-95 levels in the ACC at the early time-point whereas haloperidol is responsible for increasing the same mRNA in the medial agranular cortex, motor cortex, and somatosensory cortex [119]. *D-aspartate-oxidase* KO mice (*Ddo*^−/−^), with persistently elevated brain D-aspartate levels, exhibited a decrease in *PSD-95* expression in the motor cortex (*p* = 0.02), prelimbic cortex (*p* = 0.01), and in several following regions of caudate putamen-dorsomedial (*p* = 0.001), dorsolateral (*p* = 0.005), ventrolateral (*p* = 0.0003), ventromedial (*p* = 0.001) compared with wild type mice, suggesting that persistently elevated D-aspartate may cause complex adaptive mechanisms affecting striatal and cortical gene expression of key PSD transcripts by enhancing NMDA transmission [115]. Additionally, D-serine seems to be involved in modulating the interaction of postsynaptic SR with PSD-95, NMDAR, and other postsynaptic scaffold proteins as well as postsynaptic PSD-95 signaling. This interaction with nitric oxide synthase (nNOS) and synaptic AMPARs demonstrates the involvement of D-serine/SR with PSD-95 and NMDARs in glutamatergic synapse stability during synaptic development [137].

## 6. DISC1 a Scaffolding Protein

### 6.1. DISC1 Distribution, Structure and General Function

*DISC1* is a susceptibility gene for schizophrenia and putatively for other psychiatric disorders, initially discovered in a Scottish family with a high frequency of mental disorders [179,185,186,187,188]. DISC1 is a scaffold protein with a highly complex interactome at the PSD site and is involved in multiple brain functions relevant to the symptomatology and pathophysiology of schizophrenia [189]. Growing biological studies have indicated the relevance of DISC1 in early neurodevelopment and synaptic regulation, suggesting its role in determining multiple endophenotypes underlying major mental disorders [185]. Several reports have shown that common variants of *DISC1* are associated with endophenotypes relevant to schizophrenia, including reduced gray matter volume and poor working memory [190,191,192,193]. Post-mortem immunoelectron microscopy studies have found that a pool of DISC1 was enriched in the PSD of the human brain [110], suggesting the relevance of DISC1 in synaptic activity. Even with the limitation of KO modeling, *DISC1* KO mice may recapitulate some of the functional milestones associated with psychiatric disorders. In this framework, mice lacking *DISC1* gene fast-spiking interneurons (INTs) showed reduced average firing rates of INTs with decreased spike transmission efficacy at local pyramidal cell (PYR)-INT connections in vivo, suggesting reduced excitatory responsiveness to local glutamatergic drive as a potential mechanism of lower INT rates [194]. The *DISC1* KO mice might be considered a proxy of prodromal schizophrenia pathophysiology relevant for studying psychotic conversion from the prodromal to the full-blown psychosis phase, suitable for early intervention [195]. In this context, the chronic administration of N-acetylcysteine (NAC) has been shown to reduce the amphetamine-induced hyperlocomotion and revert the subcortical “dopamine storm” in animal modeling by acting on dopamine receptors, expression of glycogen synthase kinase 3 (GSK3), medium spiny neuron (MSN) dendritic impairments, and striatal parvalbumin (PV) density, demonstrating the preventive effect of chronic NAC treatment. These findings supported the benefit of NAC as a dietary supplement for schizophrenia prodromes but also the relevance of striatal dopamine receptors, PV neurons, and GSK3 signaling pathways as therapeutic targets for treating or preventing the pathogenesis of mental disorders [195].

Beyond PSD localization, DISC1 has been detected in the cytoskeleton along with actin filaments and girdin, which, via kinesin-based transport along the cytoskeleton, might play a role in axonal elongation [196,197]. Of interest, it has been hypothesized that truncated DISC1 might be responsible for microtubule disruption and a disorganized microtubule network [196,198,199]. DISC1 has also been localized to growth cones, detected in the hippocampus, interacting with multiple binding partners [197,199,200], such as the centrosome, which is involved in the recruitment of kendrin, dynein, and dynactin subunits, lissencephaly 1, nuclear distribution protein E homolog-like-1 (NDEL1), pericentriolar material 1 (PCM1), ninein, and coiled-coil protein associated with myosin II and DISC1. The centrosome has also been shown to interplay with cAMP-specific 3′,5′-cyclic phosphodiesterase 4B/4D (PDE4B/PDE4D), nuclear distribution protein E homolog 1, or nuclear distribution element 1 (NDE1), and Bardet–Biedl Syndrome 4, and these interactions have been implicated in microtubular asters formation, neurite outgrowth, and neuronal migration [198,201,202,203,204,205,206,207]. DISC1 has also been shown to localize to several subcellular compartments, including cilia, which have been implicated in the regulation of their formation and maintenance. In this regard, it has been reported that the knockdown of DISC1 induced a marked reduction in primary cilia and established subtype-specific targeting of dopamine receptors on the ciliary surface, suggesting a relationship between DISC1 and neural dopamine signaling [208]. Further localization of DISC1 has been found on membrane fractions, where it has been shown to interact with amyloid precursor protein (APP), crucial in the formation of cortical precursors [209,210,211], and with mitochondria present on microtubules, regulating their “ring” structures and trafficking. Moreover, interaction with mitofilin within mitochondria has been reported to be critical for the proper functioning of the electron transport chain, monoamine oxidase, and Ca^2+^ activity [212,213,214,215].

### 6.2. DISC1 Connectome and Signalosome 

DISC1 is a molecular partner in a large interactome comprising more than a hundred binding molecules, which acts as a structural and functional protein regulating multiple and parallel signaling pathways implicated in neurodevelopment diseases [216]. The function of DISC1 has been studied in human induced pluripotent stem cells (iPSCs) inoculated with an endogenous 3X-FLAG tag at the C-terminus of canonical DISC1 using CRISPR/Cas9 (clustered regularly interspaced short palindromic repeats/CRISPR-associated protein 9) [187,216,217,218,219]. In this context, DISC1 has been proven to act in a cell type- and context-dependent fashion, relevant to the neural development and integration of psychiatric disease risk factors within a set of defined molecular functions [216]. Indeed, DISC1 has been shown to regulate cell division, metabolism, and synaptic function based on its location and protein interaction in cytoplasmic compartments, such as the kinetochore, mitochondria, and pre-and postsynaptic sites, respectively [44,185,220,221]. However, most of the connections identified in the interactome have not yet been fully validated, and the functional outcome of these interactions remains to be elucidated.

**Figure 2:**
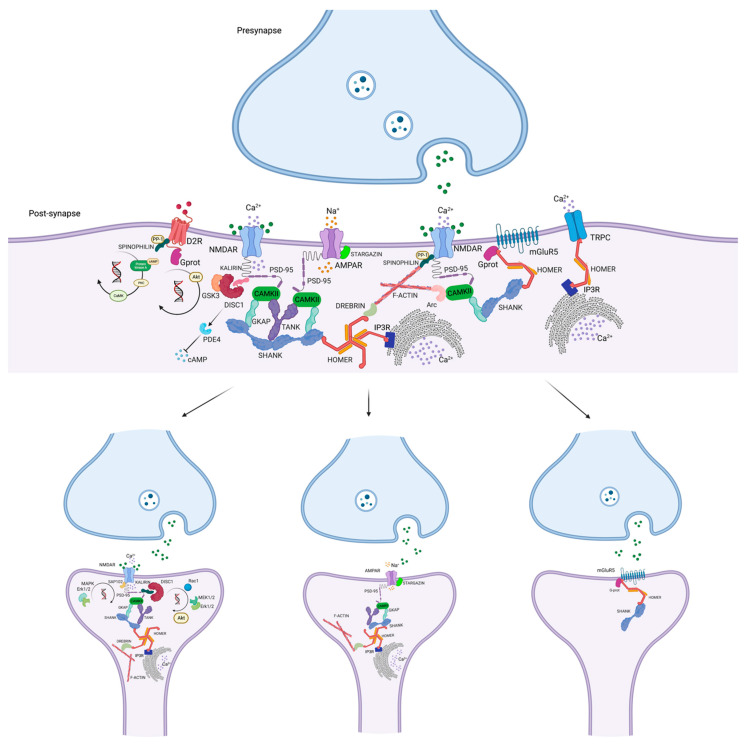
Focus on signaling at the PSD: NMDAR, AMPAR, mGluR5. Representation of transduction pathways of glutamatergic and dopaminergic receptors at the PSD. AMPAR = α-amino-3-hydroxy-5-methyl-4-isoxazolepropionic acid receptor; CAMKII = calcium–calmodulin (CaM)-dependent protein kinase II; F-ACTIN = filamentous actin; GKAP = guanylate kinase-associated protein; mGluR5 = metabotropic glutamate receptor 5; NMDAR = N-methyl-D-aspartic acid receptor; PSD-95 = postsynaptic density protein 95; SHANK = SH3 and multiple ankyrin repeat domains; TANK = TRAF family member associated NFKB activator; PP-1 = protein phosphatase 1; D2R = dopamine receptor D2; Gprot = G protein; cAMP = cyclic adenosine monophosphate; AKT = protein kinase B; PKC = protein kinase C; GSK3 = glycogen synthase kinase 3; Ca^2+^ = calcium ion; DISC1 = disrupted-in-schizophrenia 1; PDE4 = phosphodiesterase 4A; Na^+^ = sodium ion; IP3R = inositol 1,4,5-trisphosphate receptor, type 3; TRPC = transient receptor potential cation channel; SAP102 = synapse-associated protein 102; MAPK = mitogen activated protein kinases; Erk = extracellular signal-regulated kinase; Rac1 = Rac family small GTPase 1; MEK = mitogen-activated protein kinase. Created with BioRender.com; last access on 30 November 2022.

**Figure 3:**
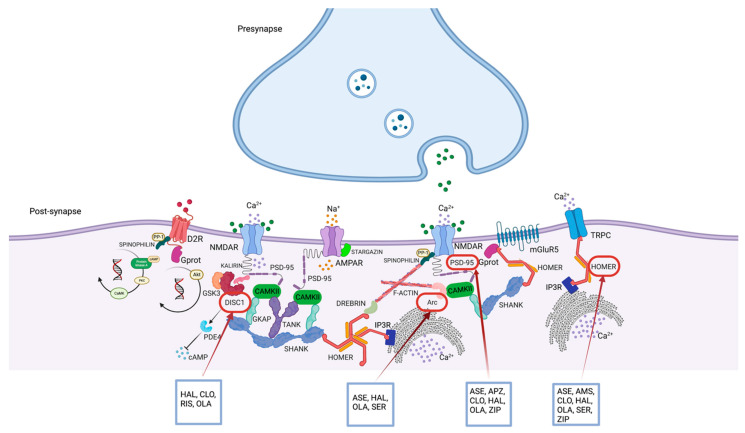
Main PSD proteins modulated by the receptor profile of different classes of antipsychotics. AMPAR = α-amino-3-hydroxy-5-methyl-4-isoxazolepropionic acid receptor; CAMKII = Calcium–calmodulin (CaM)-dependent protein kinase II; F-ACTIN = filamentous actin; GKAP = guanylate kinase-associated protein; GSK3 = Glycogen Synthase Kinase 3 Beta; IP3R = IP3 (inositol 1,4,5-trisphosphate) receptor; mGluR5 = metabotropic glutamate receptor 5; NMDAR = N-Methyl-D-aspartic acid receptor; SHANK = SH3 and multiple ankyrin repeat domains; TANK = TRAF Family Member Associated NFKB Activator; PP-1 = protein phosphatase 1; D2R = dopamine receptor D2; Gprot = G protein; cAMP = cyclic adenosine monophosphate; AKT = protein kinase B; PKC = protein-chinasi C; GSK3 = glycogen synthase kinase-3; Ca2+ = calcium ion; DISC1 = disrupted-in-schizophrenia 1; PDE4 = phosphodiesterase 4A; Na+ = sodium ion; IP3R = inositol 1,4,5-trisphosphate receptor, type 3; TRPC = transient receptor potential cation channel; HAL = haloperidol; CLO = clozapine; RIS = risperidone; OLA = olanzapine; SER = sertindole; ZIP = ziprasidone; APZ = aripiprazole; AMS = amisulpride; ASE = asenapine. Created with BioRender.com; last access on 30 November 2022.

Preclinical animal model studies in which *DISC1* isoforms were genetically deleted demonstrated morphological changes in the dendritic spine [222]. The role and specificity of DISC1 in modeling the dendritic spine were elucidated after the discovery of its binding to Kalirin7 (Kal7), a key regulator of the dendritic structure, preventing the interaction of Kal7 with Rac1, GTPase protein that is highly reactive to NMDAR activation by glutamate, thereby reducing NMDAR/NR2-dependent signaling [223] (Figure 2) (Table 1). This mechanism, mimicking disturbances in glutamatergic neurotransmission, has been frequently reported in schizophrenia, leading to the disruption of dendritic spines, and resulting in major pathological changes in brain function. Furthermore, the concept of a signalosome involving disease-associated factors, such as DISC1 and glutamate, may contribute to the multifactorial and polygenic features of schizophrenia [223]. Several lines of evidence have suggested a putative role for DISC1 in regulating fundamental events in neural development, from precursor cell proliferation and differentiation to synaptic specialization and function. Although the mechanisms leading to the regulation of gene expression are unknown, studies in mouse hippocampal neurons [224] have shown that DISC1 regulates the transcriptional activity of activating transcription factor 4 (ATF4), also known as cAMP-responsive element binding protein-2 (Creb2) [225], contributing to tumor formation. Of interest, ATF4 has been identified as a transcription factor linked to synaptic processes associated with cognitive function, and DISC1 is its most abundant binding partner in the nucleus of mutant human neurons, showing an increased connection to multiple dysregulated genes [224]. In this regard, preclinical studies identified a binding region between *DISC1* and *ATF4* in the genomic locus of PDE4D, a gene implicated in psychiatric disorders [226]. The results of this study showed that loss of *DISC1* or *ATF4* function increased the transcription of PDE4D9. Considering that PDE4D9 is increased by dopaminergic stimulation of D1-type dopamine receptors [227,228], the study results suggested that DISC1 release mediated by transcriptional repression of PDE4D9 might act as a regulator of feedback inhibition of dopaminergic signaling [224]. Considering the involvement of DISC1 in many neural functions, such as neurogenesis, neural differentiation, migration, and neurotransmitter signaling, how can changes in DISC1 structure and function affect the dopaminergic transmission, and how can they interfere with the pathophysiology of schizophrenia? Gathering together the long-recognized alterations in dopaminergic neurotransmission in schizophrenia, a surprising impact of DISC1 on dopaminergic function has been elucidated with increasing attention to its role in the dopamine D2 receptor (D2R) regulation and the response to antipsychotics [229]. In this regard, DISC1 has been shown to form a protein complex with D2R, the principal pharmacological target of antipsychotics. The D2R-DISC1 complex has been found in the brain tissue of patients with schizophrenia, promoting GSK3 signaling and inhibiting D2R internalization (Figure 2 and Figure 3). Su and colleagues demonstrated that the *DISC1* variant R264Q, located within the D2R-binding region, was shown to increase DISC1 affinity for D2R by promoting GSK3 activity, suggesting a possible mechanism by which this common polymorphism could influence aspects of brain function relevant to schizophrenia, which might be explored for the development of novel drug treatments [229]. Concerning other neurotransmitter signaling pathways in which DISC1 is implicated, it should be noted that *DISC1* heterozygous mutant mice show elevated levels of PDE4 associated with synaptic abnormalities in the cortex, hippocampus, and the whole brain, with social and cognitive behavioral deficits [230]. In this framework, DISC1 has been shown to bind PDE4 family members that negatively regulate adenosine 3′,5′-cyclic monophosphate (cAMP) signaling, negatively influencing synaptic plasticity [231] (Figure 2). In this view, the PDE4 pathway may represent pathological molecular signatures in human neurons derived from patients with schizophrenia and other major mental disorders and potentially considered a target for drug development. An intriguing relationship between PDE4 signaling and the *DISC1* mutation has been documented with potential therapeutic implications. Specifically, cortical neurons differentiated from iPSCs derived from patients with schizophrenia and carrying a deletion in the *DISC1* gene showed a reduction of synaptic vesicle protein (SV2^+^) puncta number after four weeks of neural differentiation [232], associated with an increase in PDE4-related signaling [230]. Some studies showed that pharmacological inhibition of PDE4 has proven to restore synaptic transmission and social and cognitive behavioral alterations [233,234]. Collectively these findings suggested that dysregulation of PDE4-cAMP signaling might be a potential convergent patho-molecular mechanism underpinning schizophrenia and other mental disorders and could provide a target for future drug development [230]. 

### 6.3. DISC1 and Schizophrenia: Human Common and Ultrarare DISC1 Variants

A growing number of biological studies have focused on the *DISC1* gene, a well-established genetic risk factor in a spectrum of psychiatric disorders, supporting the connection between common and ultrarare *DISC1* variants and their structural and functional implications associated with major mental illnesses [187]. Three common *DISC1* gene variants associated with behavioral disorders have been discovered: L607F, S704C, R264Q [193,235], and several rare and ultrarare variants: G14A, R37W, S90L, R418H, T603I, S209R, R338Q, T754,S and P758R [236,237,238]. It has been reported that the common variants S704C and R264Q may slightly increase the risk of developing schizophrenia, whereas the rare and ultrarare variants are associated with a 2% risk of schizophrenia [193]. Reduced gray matter in the superior frontal gyrus, anterior cingulate cortex, and left supramarginal gyrus was found in F607 allele carriers compared with L607 homozygotes, as well as suffering from more severe positive symptoms in schizophrenia, providing a potential mechanism by which DISC1 could confer an increased risk for schizophrenia [239,240]. Of interest, a recent report showed that schizophrenia patients with severe positive symptoms who do not respond to antipsychotic treatment showed a reduced superior frontal metabolism [241]. In particular, in vitro studies have found that the F607 variant is associated with a reduced release of noradrenaline, and a reduced ability of DISC1 to interact with the PCM1 protein of the centrosome, resulting in defects in mitochondrial trafficking [214,242]. Furthermore, carriers of the F607 allele also express lower levels of a *DISC1* splice variant expressed in patients with schizophrenia [243]. The S704/C704 variant has been associated with structural (including volume and integrity of gray matter) [244,245] and functional abnormalities of the hippocampal and para-hippocampal regions [190,246], severe positive symptoms in patients with schizophrenia [247], and further DLPFC activity during a working memory task [248]. Studies on the effects of R264Q have been limited to a modest impact on cortical thickness in the lateral occipital gyrus [239] while evidence of modifications caused by L607F and S704C has emerged [236]. At the molecular level, as with L607/F607, the 704 amino acid variation has also been implicated in altered PCM1 localization and *DISC1* expression [243,249]. In addition, the C704 allele has been associated with reduced activity of extracellular signal-regulated kinase 1 (ERK1) and serine/threonine kinase RAC-alpha (AKT) (Figure 2), altered DISC1 binding affinity for NDE1 and NDEL1, and a change in DISC1 oligomeric status [206,244,250,251], relevant for schizophrenia.

### 6.4. DISC1, a Drugable Molecule?

DISC1 is a multifunctional scaffold protein that interacts with several proteins involved in neuronal migration, neurite outgrowth, cytoskeleton modulation, and signal transduction [252] whose interactome has been an attractive target for potential therapeutic interventions [253]. Chronic administration of the atypical antipsychotics olanzapine and risperidone has been shown to increase *DISC1* mRNA expression in the frontal cortex of mice, whereas haloperidol and clozapine had no effect (Table 2). Furthermore, olanzapine also has been shown to increase *DISC1* mRNA expression in the hippocampus similar to risperidone [254] (Figure 3). In clinical trials, *DISC1* missense variants have been found in patients affected by schizophrenia with a form of ultra-resistance to treatment, suggesting that *DISC1* variants may have a functional influence on the mechanisms of action of antipsychotics and treatment response [255]. In this regard, it has been reported that repeated oral administrations of clozapine, but not haloperidol, in the dominant-negative form of disrupted-in-schizophrenia 1 (DN-DISC1) transgenic mice treated with polyinosinic:polycytidylic acid (polyI:C), were able to improve cognitive impairment in the animal model. This study postulated that the subsequent increase in DISC1 may impact modulation of synaptic spines as a potential mechanism of action of the atypical antipsychotics [256] (Figure 3). The main reasons currently hindering the development of an effective small molecule targeting DISC1 are mainly the lack of knowledge of the multiple forms of splicing, 3D structure, and a large number of interacting proteins (about 200) [12,187]. The potential impact on AKT signaling and the growing evidence for a role in dopaminergic and glutamatergic signaling have placed the DISC1 pathway as a promising but challenging target for future pharmacological development [187].

## 7. The Homer Proteins at the PSD

### 7.1. Homer1: Distribution, Structure, and General Function

A multifunctioning and highly interacting class of proteins at the PSD, the Homer1, were discovered and cloned in a series of experiments reputed to unveil the molecular determinants of mGluR type I signaling [257] at a glutamatergic synapse [12,258,259]. Even if detected in several different tissues (i.e., skeletal muscle, heart), the largest distribution is the cerebral one. In the brain, the highest concentration has been reported (in decreasing order) in the striatum, cortex, and the hippocampus [260,261,262,263]. At least two isoforms of Homer1 proteins have been described: constitutive (Homer1b/c, Homer2a/b, Homer3) and inducible (Homer1a and Ania-3). The long form Homer1b/c is characterized by enabled/vasodilator-stimulated phosphoprotein homology (EVH1) sequence at the N-terminus, and a coiled-coil forms at the C-terminus that allows the single molecules of Homers to interact with each other. It forms a sea-star-like macromolecule, whose free-standing EVH1 terminal may interact with multiple intracellular targets included among others: PSD proteins (i.e., PSD-95, Shank1) [8,260,264,265,266]. Homer1 plays an important role in working memory [267,268], executive functions [269], and regulation of cortical hyperexcitability, and it has been hypothesized to play a pivotal role in dopamine-glutamatergic neurotransmission, relevant in the pathogenesis of schizophrenia [270,271,272]. Moreover, Homer1 has multiple functions including modulation of mGluR type I trafficking, regulation of Ca^2+^ signaling, control of LTP, growth of dendritic spines, and control over synaptic plasticity [8].

### 7.2. The Homer Connectome and Signalosome

Homer1s are capable of dispatching intracellularly multiple convergent or divergent signaling, this feature may be the result of the different isoforms, the constitutive or immediate early-gene related functions, as well as the sophisticated Homer interactome [8,273]. Homer1-interaction partners are reported in UniProt [274]. In fact, among the PSD proteins, Homer1 is a key component of the dendritic spine and a regulator of neuronal signaling [275,276,277]. A principal implication of Homer1 in cellular signaling is the modulation of glutamatergic transmission mainly related to mGluR type I, due to the role of glutamate in schizophrenia pathophysiology makes the Homer1 family of potential relevance both for the structure and function of the dendritic spine at a glutamatergic synapse (Table 1). The assembling/disassembling of Homer1 and its interacting partners is probably the most evident consequence of glutamate transmission effects on Homer-related signal transmission. One interaction that has been recently highlighted for a robust association between glutamate function and schizophrenia is the one between Homer1 and insulin receptor substrate type53 (IRSp53), aka BAR/IMD domain containing adaptor protein 2 (BAIAP2). The latter is a scaffold protein located at the PSD of glutamate synapse that bridges membranes with the cytoskeleton in actin-filled protrusions and is believed to be implicated in synapse development. In addition, the IRSp53 and Homer1 interaction is probably mediated through PSD-95 [278]. The induction of Homer1a by synaptic stimuli impacts intracellular Ca^2+^ oscillations through the regulation of the coupling of long Homers with IP3R, transient receptor potential channels (TRPCs) (Figure 2), and ryanodine receptor (RyR) [279].

### 7.3. Homer, Schizophrenia, and Antipsychotic Treatment

Three studies have investigated Homer’s expression in the post-mortem brain of schizophrenia patients with a focus on the cortex and/or hippocampus. A decrease in Homer levels was detected in the cortex and hippocampus of schizophrenia patients compared to HC with no separation between the two major isoforms [11]. A study conducted on the CA1 region of the hippocampus (20 schizophrenia patients and 20 HC brains) has shown a reduction in the protein levels of the long form Homer1b/c (−43%) and an increase of the short form Homer1a (+25%) with a ratio Homer1a/Homer1b-c >1 [111]. Changes were also reported in several proteins interacting directly or indirectly with Homer1-included PSD-95 [280]. Finally, utilizing products determined with denaturing high-performance liquid chromatography and analyzed by polymerase chain reaction, it was demonstrated that the allele frequency of single nucleotide polymorphisms (SNPs) in a DNA pool of 368 schizophrenia patients and 368 HC, *Homer1-IVS4+18A>G* (statistically significant with individual genotyping, *p* = 0.01) were associated with schizophrenia, identifying a plausible candidate for the phenotype of the disease [281]. Preclinical studies in rats have shown that *Homer* expression was changed by the administration of different antipsychotics (Table 2). Specifically, *Homer1a* was significantly induced by haloperidol in dorsomedial, dorsolateral, ventromedial, ventrolateral caudate-putamen, and NAc [106,108,112], and by asenapine and olanzapine in dorsolateral caudate-putamen [112]. Furthermore, olanzapine induced *Homer1a* gene expression in the caudate-putamen regions and in the nucleus accumbens shell, whereas asenapine induced it in the ACC, medial agranular cortex, motor cortex, dorsolateral caudate-putamen, and NAc [112,113]. *Homer1a* was induced by tergulide in ventrolateral caudate-putamen [118]. Additionally, tergulide also reduced *Homer1b* in the ventrolateral caudate-putamen, ventromedial caudate-putamen, and shell of NAc [118]. Preclinical studies demonstrated that ketamine increased *Homer1a* expression in the insular cortex, and decrease *Homer1b* in the motor cortex, dorsolateral caudate-putamen, ventromedial caudate-putamen, and medial agranular cortex [114,117]. In acute administration of clozapine, *Homer1a* was increased in all caudate-putamen subregions, in the NAc, and medial agranular cortex. In addition, clozapine and ziprasidone both induced *Homer1a* in somatosensory cortex, and insular cortex. The chronic administration of ziprasidone increased *Homer1a* only in the NAc and dorsolateral and ventrolateral caudate-putamen, and even induced *Homer1b* in dorsolateral caudate-putamen. During chronic clozapine treatment *Homer1a* expression was induced only in the NAc, suggesting that *Homer1a* preserves its expression profile after chronic antipsychotics [119]. Haloperidol also induced *Homer1b* in the ACC, motor cortex, medial agranular cortex, and insular cortex. Furthermore, *Homer1b/c* expression was increased more by asenapine than by haloperidol in the insular cortex and somatosensory cortex [116]. In this context, the impact of amisulpride on gene transcripts when administered in naïve rats compared with rats pretreated with antipsychotics demonstrated differential effects in the cortex and striatum by increasing *Homer1a* levels in the NAc nucleus and inducing *Homer1b/c* in striatal regions more than haloperidol [116]. These results are suggestive of a differential neurobiological impact on PSD transcripts induced by antipsychotics.

## 8. Other PSD Proteins

### 8.1. The Role of PSD Transmembrane AMPAR Regulatory Proteins in Schizophrenia: Stargazin

The transmembrane AMPAR regulatory proteins (TARPs), such as stargazin, were demonstrated to be crucial for the AMPAR subunits, regulating their both receptor trafficking and gating [153] (Figure 2). Stargazin showed a positive allosteric modulation of AMPARs, promoting the AMPAR surface expression and localization, exerting a regulatory role of intracellular channel conductance [282]. On the surface of the PSD, AMPAR binds to stargazin, connecting the intracellular proteins containing PDZ domains, such as PSD-95, stabilizing the activated state of AMPAR [283,284]. Indeed, in neurons that do not express stargazin, the majority of AMPARs fail to reach the dendritic surface and a substantial proportion of AMPARs, which are intracellularly retained, exhibit immature glycosylation crucial for the efficacy of synaptic transmission in the brain [285]. Preclinical studies on hippocampal slices suggested that synaptic recruitment and activation of AMPAR are regulated by PSD-95 via stargazin, which is involved in the extra-synaptic expression of AMPAR [286] and interactions with PSD-95 for stabilization and NMDAR-dependent trafficking [287]. Several authors have pointed out that LTP and long-term depression (LTD), the most extensively studied forms of long-lasting synaptic plasticity regarded cellular mechanisms underlying learning and memory, are associated with AMPAR recruitment [288] and trafficking [289]. Preclinical studies have reported that while showing normal localization of NMDARs, PSD-95 mutant mice exhibit deficits in LTP, LTD, and spatial learning, suggesting that PSD-95 may play a critical role in coupling NMDARs to downstream transduction pathways [290]. Furthermore, PSD-95 also appears to be indirectly linked to AMPARs through stargazin interaction [286]. The bidirectional interconnection between PSD-95 and stargazin mediates the targeting of AMPARs to the synaptic membrane, and, therefore, it is conceivable that a functional link between NMDARs and AMPARs occurs via the PSD-95-stargazin complex, potentially dysregulated in the pathophysiology of schizophrenia. In a recent post-mortem study of patients with schizophrenia, Benesh and collaborators suggested that associations between stargazin and AMPA subunits are abnormal, potentially affecting the forward trafficking or synaptic stability of subunit of glutamate GluA1-containing AMPARs. This study provides evidence of altered interactions with trafficking proteins that contribute to glutamate dysregulation in schizophrenia [291]. AMPARs and their modulators, such as TARPs, mediate most of the fast synaptic transmission in the mammalian brain and spinal cord, representing attractive targets for therapeutic strategies [92], also with a role as anticonvulsants or neuroprotectants [292] (Table 1). Moreover, the trafficking and gating properties of AMPARs may be amplified by these TARP proteins expressed in localized brain regions, including the forebrain and hippocampus [293]. However, bioavailability could be a frequent failure of small molecules that target PSD proteins, such as TARPs, that amplify AMPAR pharmacology by altering the rapid excitatory properties of glutamate [12]. Of interest, the potential strategy of adding compounds to antipsychotics that modulate TARPs requires further investigation as a possible approach in schizophrenia treatment [12]. Remarkably, genes encoding for PSD proteins have been considered promising candidates for the identification of new treatments in diseases where PSD dysfunction underpins a genetic framework of vulnerability, such as schizophrenia. It should be noted that the calcium channel gamma-2 subunit gene, which codes for stargazin, has been considered a pharmacological target gene [294]. In light of this, several findings have contributed to highlighting the association of PSD genes related to neurodevelopmental disorders (especially schizophrenia and ASD), including whole-genome analyses linking multiple TARP loci to childhood epilepsy, schizophrenia, and bipolar disorder where, chaperones AMPA receptors emerge as crucial in excitatory synapses, implicating both in signal transduction and in neuropsychiatric disorders [295]. As a matter of fact, recent GWAS findings, proteomic, post-mortem, neuroimaging, and genetic analyses, as well as preclinical models, have suggested a relevant role of PSD genes in the pathophysiology of schizophrenia [8,11,23,24,171,296,297]. 

### 8.2. Arc and PSD

#### 8.2.1. Arc Distribution, Connectome, and Signalosome

The activity-regulated cytoskeleton-associated protein (Arc) (also known as Arg3.1, and KIAA0278) is an immediate early gene (IEG) encoding for a protein consisting of a coiled-coil domain at the N-terminus, multiple endocytic protein binding domains, and a homology domain with spectrin, a cytoskeletal protein, at the C-terminus [298]. *Arc* gene presents a DNA sequence normally found in the RNA of the human immunodeficiency virus, responsible for forming virus-like particles [299]. Thanks to its structure, *Arc* mRNA is able to link Arc protein and form a capsid containing *Arc* mRNA. These structures are encapsulated in the extracellular vesicles that cross the neuron membrane to other cells, such as microglia or other neurons, in a spatial/temporal specific manner, relevant for Arc activity, including synaptic plasticity, learning, memory consolidation, and behavior experiences [300,301,302]. At the PSD site, *Arc* mRNA localizes in the PSD-95-NMDAR complexes with kinesin motor complex and other proteins including fragile X mental retardation protein (FMRP) and Pur-alpha [303]. At this site, Arc interacts directly with an inactive form of CaMKIIβ targeting the spine as actin-rich dendritic spines, relevant for synaptic plasticity [304] (Figure 2). Given its centrality in synaptic architecture, Arc is highly conserved in vertebrates and is specifically expressed in the nucleus and dendrites, and at the PSD of cortical and hippocampal glutamatergic neurons [305]. *Arc* is the only IEG that moves through the dendritic arborization after its expression, differently from the others that remain localized in the cell body [306]. Its transcription is inhibited by AMPAR, which regulates its levels after about five minutes of its induction by various stimuli, including seizures in the hippocampus, LTP, and LTD [307,308,309]. The C-terminus of Arc is involved in the regulation of cytoskeleton structure via interaction with F-actin [305], microtubules, and microtubule-associated protein 2 [310]. Furthermore, Arc is able to maintain the phosphorylation of cofilin, the actin depolymerization factor, resulting in an increase of F-actin [311]. This process is responsible for an increase in the proportion of thin and filopodia-like protrusions and density of spines in neurons of the hippocampus as demonstrated in a comparison of wild type and *Arc* KO mice [312]. Concomitantly, mature and mushroom-shaped spines are found in the same animal model indicating a plausible negative influence of *Arc* on spine maturation [312]. Moreover, a decrease in spine density seems to be related to *Arc* aberrant expression related to NMDAR hypofunction [70], emphasizing its role in regulating dendritic spine density and morphology [313], relevant for schizophrenia (Table 1). The link between Arc expression and glutamate neurotransmission seems to be related also to AMPAR function through its association with Arc’s endocytic protein-binding domains [298,314]. In fact, Arc is also involved in modulating AMPAR trafficking through its translocation into the nucleus by regulating transcription and homeostatic plasticity [314,315,316,317]. On the other hand, NMDAR, mGluR type I, Tropomyosin receptor kinase B, and muscarinic acetylcholine receptor promote Arc transcription, converging on protein kinase A (PKA), protein kinase C (PKC) [318], and ERK [319].

#### 8.2.2. Arc in Schizophrenia and Antipsychotic Modulation

Changes in *Arc* expression seem to be associated with several mental disorders, including major depressive disorder and schizophrenia [320,321,322]. Furthermore, aberrant expression of *Arc* could be relevant for cognitive impairment, fragile X mental retardation syndrome, as well as its reduced ubiquitination may occur in Angelman syndrome [322,323,324]. Kirov and colleagues, using a systematic analysis of synaptic protein complexes merging with novel copy number variants (CNVs) datasets, identified de novo mutations in the PSD proteome by enrichment for NMDAR and the Arc protein complex [89]. This study indicated that defects in postsynaptic signaling of NMDAR and Arc might play a significant role in the pathogenesis of schizophrenia [89]. Additionally, Arc is involved in the interaction between γ-secretase and trafficking endosomes relevant for the processing of APP to β-amyloid peptide, resulting in an increase in the levels of the latter related to Alzheimer’s disease [325,326,327,328]. Concerning the neurobiological basis of schizophrenia, several preclinical studies have demonstrated the involvement of Arc in dopamine-glutamate interaction within the PSD and antipsychotic modulation, relevant for synaptic plasticity [329,330,331] (Figure 3).

Both typical and atypical antipsychotics can modulate *Arc* expression [332,333]. In this regard, typical antipsychotic haloperidol is responsible for an *Arc* increase in the NAc and striatum after 1h from the treatment, while the atypical antipsychotic clozapine reduces Arc levels in the thalamus and hypothalamus after 6h of treatment [334]. Another study evaluated the effects of the same drugs after acute and chronic treatment on the Arc protein levels showing an increase in the caudate after haloperidol administration in both settings but no effect after clozapine administration [335]. Furthermore, haloperidol increased its levels in the NAc core following acute treatment different from clozapine, which increases its levels in the shell of the NAc after acute treatment [335]. Clozapine was shown to decrease Arc protein levels in the mPFC and cingulate cortex after both acute and chronic administration [335]. Haloperidol induced also an increase of *Arc* in the whole striatum [106,107] except for the NAc shell compared with vehicle and sertindole [109]. A preclinical study demonstrated *Arc* increased levels in the striatum at 30, 60, and 120 min after haloperidol injection, while olanzapine was responsible for an increase only at the 30 min time point. At the same time, both haloperidol and olanzapine reduced *Arc* levels in the frontal cortex at 60 min [336]. Further preclinical evidence showed that haloperidol significantly increased *Arc* levels compared with amisulpride in the striatum [333]. Additionally, caffeine can modulate the effect of *Arc* mRNA and protein levels induced by haloperidol; in fact, the co-administration of caffeine and haloperidol reduced the *Arc* expression in the striatum and cortex compared with haloperidol [108] (Table 2). Finally, pretreatment with atypical antipsychotics 1 h before the administration of phencyclidine (PCP), which mimics psychotic symptoms in murine animal models, significantly reduced the *Arc* PCP-induced increase in the mPFC and NAc, suggesting a crucial role of antipsychotics in Arc modulation, relevant for therapeutic effects [337].

### 8.3. Neuregulin

The trophic factor Neuregulin 1 (*NRG1*, also known as heuregulin1 or neu differentiation factor) is a member of the epidermal growth factor (EGF) family which is involved in the inhibitory control of executive functions including working memory [338,339]. *NRG1* is one of the four members of the *NRG* gene family characterized by an EGF-domain whose target is represented by ErbB receptors implicated in learning, memory, and other higher brain functions [340]. Multiple lines of evidence have pointed to the NRG1–ErbB4 signaling in parvalbumin-positive (PV+) basket cells as responsible for the reduction of cortical excitability [341] through the stimulation and release of the inhibitory transmitter γ-Aminobutyric acid (GABA) [342]. The NRG1 EGF-like domain is involved in the regulation of gamma oscillations [338], pyramidal cell excitability [339], seizure activity [343,344,345], and synaptic plasticity [346,347,348,349]. In addition, *NRG* is also responsible for the regulation of neural synchrony of local field potentials in the ventral hippocampus and PFC [350]. The activation of ErbB4, through the NRG1–EGF-like domains mediated phosphorylation, results in the activation of several intracellular cascades relevant for the incorporation of AMPA receptors into postsynaptic membranes and NMDAR regulation in interneurons, potentiating excitatory neurotransmission as demonstrated by preclinical study [57,80,123,351,352,353]. Therefore, the enhanced stimulation of ErbB4 on the cell surface could be responsible for increased downstream molecular signaling at the PSD level, facilitating receptor dimerization and efficient *NRG* signal transduction [78]. *NRG* signaling is also directly related to PSD-95, increasing its levels near excitatory synapses, and regulating synaptic molecular composition through stimulation of ErbB4 receptors and the subsequent activation of mitogen-activated protein kinase (MAPK) [80,122,352,354,355,356,357,358]. Specifically, the NRG1/ErbB4 pathway was seen to interact with PSD-95 and NMDAR, and that chronic administration of MK-801, induced a short-term potentiation of the functional interaction between NRG1/ErbB4 and NMDAR in the PFC and hippocampus of the rat, along with anxiety-like behavioral abnormalities, providing further evidence for cross-talk between the two signaling pathways [79]. Furthermore, Shi and collaborators recently proposed that the downregulation of the NRG1-ErbB4-mediated PV+ interneuron signaling could reactivate cortical plasticity for information processing, attention, and cognitive flexibility severely impaired in neurodevelopmental psychiatric disorders, including schizophrenia [340]. Furthermore, another mechanism of NRG1/ErbB4 interactions to promote dendrite growth in mature interneurons is mediated by Kal7, whose encoding gene *KALRN* has been associated with schizophrenia [75] [359] (Table 1). Moreover, a recent study evaluated several proteins of PSD and discovered 10 rare missense mutations in PSD protein-related genes, including *NRG1* as susceptibility factors for schizophrenia [10] (Table 2). These data are coherent with several preclinical studies pointing to the involvement of the NRG1/ErbB4 pathway in the pathophysiology of schizophrenia as demonstrated by the perinatal administration of phencyclidine (PCP) to rats, which models schizophrenia-like symptoms [124]. In particular, the NMDA receptor antagonism exerted by PCP in the early phases of neurodevelopment may affect NRG1/ErbB4 expression [124]. Of interest, it has been shown that ErbB4 may modulate extracellular dopamine through the p38 MAPK signaling pathway, suggestive of the role of *NRG* in regulating the dopamine pathway relevant as a pharmacological target for psychotic disorders [358].

### 8.4. Synapse-Associated Proteins Family

#### 8.4.1. Synapse-Associated Proteins Spatial and Temporal Distribution at the PSD

Dlg or SAPs are scaffold proteins, members of the MAGUK superfamily, which exert essential functions in the regulation of synaptic plasticity and metaplasticity processes [360]. As all the MAGUK proteins, SAPs are characterized by the co-expression of an Src homology 3 (SH3) domain, one to three PDZ motifs, and a GUK domain which lacks catalytic activity and is mainly involved in mediating protein–protein interaction [360]. Structural differences have been highlighted between SAPs with SAP102 lacking the N-terminus cysteines and L27 domains that are required for the synaptic clustering of SAP-90/PSD-95 and SAP97 [361]. Otherwise, the SH3 and GUK, but not PDZ, domains are required for SAP102 stabilization at spines [74]. SAP102 and SAP97 are encoded by *DLG3* and *DLG1* genes, respectively, and both molecules are expressed at the PSD level, where they serve as a bridge between membrane receptors and the intracellular cytoskeleton [362,363]. Even though it is well-defined that MAGUKs represent key components at the neuron–neuron interface, the specific SAPs interactome is highly challenging to define due to the presence of multiple motifs and, thus, several interaction partners, including scaffold proteins, cytoskeleton components, voltage-dependent channels, and neurotransmitter receptors [364,365,366,367]. However, significant interactions have been detected between the PDZ domains of SAP102 and SAP97 with the C-terminus tail of the NMDAR subunits NR2B and NR2A, respectively [362,368]. The different interactome exhibited by SAP102 and SAP97, together with a different time-dependent expression pattern, accounts for different functional tasks in terms of PSD organization and downstream signaling cascades, as discussed below. MAGUKs have been implicated in the time-dependent clustering of glutamate and other neurotransmitter receptors during the neurodevelopmental period as well as in the setting of LTP and LTD [364]. SAPs have been shown to regulate NMDAR trafficking and membrane expression by the direct interaction between NR1 and NR2 subunits with MAGUKs’ PDZ domains [364]. Moreover, the membrane surface exposure of NMDAR subunits has been found to depend on SAPs temporal patterns of expression [369]. Specifically, SAP102 expression is more pronounced during the early neurodevelopmental phases and decreases rapidly in later stages, concurring with an increase in SAP90/PSD-95, PSD-93, and SAP97 levels [369]. Along with these changes, the shift from SAP102 to PSD-95 at the PSD couples with a reduction in NR2B expression and an increase in NR2A levels [369]. In fact, NR2B has been demonstrated to preferentially interact with SAP102 whereas NR2A better binds to PSD-95 [369]. The switch between different patterns of expression of scaffold proteins and NMDAR subunits is required for fine-tuning the plastic processes that occur during the neurodevelopmental period probably by differently recruiting signaling molecules necessary for proper synaptogenesis and spines maturation [370]. SAPs proteins have also exhibited the capability to differently regulate intracellular signaling pathways. Of interest, Ca^2+^/CaMKII has been shown to bind and phosphorylate SAP97, modulating AMPAR activity [371]. Otherwise, SAP102 is essential to couple NMDAR to the MAPK/ERK pathway [372] (Figure 2). In fact, SAP102 KO mice displayed an elevation of phospho-ERK protein levels, which were attenuated in response to NMDA stimulation, and responsible for impairment in LTP processes, resulting in cognitive alterations of flexibility and spatial learning [372].

#### 8.4.2. Synapse-Associated Proteins in Psychotic Disorders: Clinical and Pharmacological Implications

Considering their relevant role in the organization of the PSD and NMDAR subunits, it is not surprising that several studies, mostly conducted on post-mortem brains, have explored the putative impact of SAP102 and SAP97 alterations on schizophrenia pathophysiology. In a cohort of 34 patients with schizophrenia, SAP97 protein levels were found to be reduced to less than half in the PFC, while SAP102 levels decreased in the hippocampal area compared with HC [142] (Table 2). In the same cohort, the correlation between SAP97 and GluR1 levels in the PFC was found in schizophrenia patients but not in HC, suggesting qualitative differences in the interaction of AMPAR with scaffold proteins [142]. The mRNA expression of SAP102 was reduced in the striatal region of post-mortem brains extracted from patients affected by severe psychiatric diseases, such as schizophrenia, bipolar disorder, and major depression, whereas no changes were observed in the DLPFC and cingulate cortex [133,145]. In another study, SAP102 transcripts levels were decreased in the hippocampus of bipolar disorder I, but not schizophrenia patients, accompanied by a reduction in the expression of NR1 and NR2A mRNA [146]. Moreover, in the PFC of patients affected by bipolar disorder, a decreased SAP102 mRNA expression was detected within small cells of layer II and large cells of layer III [143]. No alterations in SAP-102 protein levels were observed in the dopaminergic neurons of the substantia nigra pars compacta (SNc), as well as in thalamic tissues of subjects with schizophrenia [144,147]. Even preclinical studies explored the relationship between SAP proteins and psychotic disorders. Precisely, in a brain-specific conditional SAP97 KO model, mice exhibited male-specific cognitive impairments and female-specific motor deficits, along with enrichment for schizophrenia-related genes in the differential gene expression set [97]. Furthermore, haloperidol administration failed to mimic SAP97 changes in rats, suggesting its plausible primary alteration in schizophrenia patients [142]. Overall, these findings highlight the relevance of SAPs proteins in the pathophysiology of psychotic disorders probably through the regulation of glutamatergic receptors at the PSD. 

## 9. The PSD Interactome in Schizophrenia: Actin and Drebrin Interacting with the Main Scaffolding/Adaptor Protein and Receptors

### 9.1. Role of Actin Cytoskeleton in Dendritic Spine Morphogenesis and Implications in Schizophrenia

Other molecules that play an important role in the structure and function of the PSD are cytoskeletal proteins, such as actin, which interacts with numerous scaffold molecules to spatially organize the PSD [26,36] contributing to a dynamic structure [37]. Indeed, the dendritic spine is constantly changing morphology through the regulation of actin dynamics influenced by the activity of glutamate receptors [373]. Analysis of the spatio-temporal reorganization of postsynaptic substructures during LTP at the level of individual dendritic spines enables distinguishing three sequential phases of protein distribution. In the initial phase, the actin cytoskeleton is readily remodeled while the active cofilin is transported to the spine. Subsequently, a stabilization phase enables the cofilin to form a stable complex with F-actin. The third phase is the remodeling of the PSD by consolidating the position and expansion of the spine [374]. The dynamism of spine morphology is driven by the high concentration of the actin cytoskeleton in spines. Filopodia, which are thin, headless protrusions, are thought to be precursors of dendritic spines, while the drebrin, a protein that binds spines to F-actin, is responsible for the recruitment of F-actin and PSD-95 into filopodia. These components regulate spatially and temporally the morphogenesis of spine [36]. However, the initiation of spine morphogenesis precedes the synaptic assembly of PSD-95 [375] as demonstrated by mutant mice lacking PSD-95 that had normal spine morphology [290]. On the other hand, disruption of the actin cytoskeleton during spine morphogenesis results in the global disassembly of synaptic structural elements [376], suggesting its crucial role in governing spine morphogenesis. The involvement of F-actin in the structural plasticity of mature spines has already been highlighted. However, some authors have hypothesized that actin reorganization could be driven by a local increase in Ca^2+^, triggered by the activation of NMDAR and voltage-gated Ca^2+^ channels, providing the molecular basis for activity-dependent actin remodeling [377]. Furthermore, in vitro studies of neuron hippocampal cultures exposed to prolonged NMDAR activity have been shown to induce the disappearance of F-actin at synapses [378]. Moreover, these findings reported that NMDAR-induced loss of actin at spines was time- and concentration-dependent and was blocked by NMDAR antagonists, with a similar effect exerted by L-glutamate, AMPA, and ionomycin, but not by agonists of L-type calcium channels or mGluR. However, the effect of NMDAR activity on local actin assembly was found to be attenuated by pretreatment with an actin-stabilizing compound or an antagonist of the calcium-dependent protein phosphatase calcineurin, suggesting that the actin-mediated stability of the synaptic structure is disturbed by intense glutamate receptor activity and that calcineurin blockers may be useful in preventing such destabilization [378]. It has been hypothesized that in the adult brain actin is responsible for rapid changes in dendritic spines by influencing the function of glutamatergic receptors and, in turn, being influenced by them. Therefore, the actin cytoskeleton is emerging as a crucial mediator in signaling and anatomical plasticity of excitatory synapses [373]. Preclinical studies have suggested a role for actin in experience-modifiable changes in the shape of dendritic spines [379] that have been proposed as the molecular basis for alterations in synaptic connectivity thought to underlie learning and memory [380]. Recent studies have suggested that neuronal encoding of memories may be associated with the dynamic formation and elimination of spines that may be dysfunctional in various pathological conditions [379]. Post-mortem studies have found a reduced density of dendritic spines in the DLPFC of patients with schizophrenia, suggesting that the molecular mechanisms concurring with these alterations are the molecular cascades that contribute to the regulation of the actin cytoskeleton with a potential role in the pathophysiology of schizophrenia [381]. In addition, dysgenesis of dendritic spines is a neuroanatomical finding highly represented in several psychiatric disorders, including intellectual disability, ASD, and schizophrenia [382]. Among the most recently identified synaptic proteins, growing interest has been focused on p140Cap, an adaptor that localizes on dendritic spines contributing to the regulation of synaptogenesis, synaptic transmission, and synaptic plasticity [383,384,385]. Multiple lines of evidence have shown that p140Cap KO mice exhibited cognitive deficits, impaired LTP, LTD, and immature filopodia-like dendritic spines. Specifically, p140Cap KO mice showed a defect in LTP and LTD, with reduced object recognition, suggestive of alterations in memory consolidation and cognitive retrieval [385]. A number of p140Cap interacting proteins have been identified in the brain, such as SNAP-25 [384,386], PSD-95 [87], and cortactin, an F-actin-binding protein that regulates actin cytoskeleton organization [387], suggesting that p140Cap acts as a hub for PSD complexes relevant for psychiatric and neurological disorders. Interactors of p140Cap converge on key synaptic processes, including transmission across excitatory synapses, actin cytoskeleton remodeling, and cell–cell junction organization. Remarkably, the interactome of p140Cap and its co-expression network show strong enrichment in genes associated with schizophrenia, ASD, bipolar disorder, intellectual disability, and epilepsy, supporting that synaptic dysfunction is a common biological feature in cerebral diseases [388].

### 9.2. Drebrin in Synaptic Dysfunction and Neuropsychiatric Implications

Dendritic spines receive most of the excitatory synapses in the postsynapse, and their morphological plasticity plays a nodal role in higher brain functions, such as learning and memory. Drebrin is a key modulator of the actin cytoskeleton in neuronal cells, critical for synaptic plasticity, neuritogenesis, and neuronal migration by orchestrating a cross-talk between actin and microtubules. Studies have shown that decreased drebrin levels are a hallmark of multiple neurodegenerative disorders [389]. Loss of drebrin from dendritic spines has been demonstrated to be a pathognomonic feature of synaptic dysfunction associated with neurological disorders accompanied by cognitive deficits [36]. Preclinical in vivo immunoelectron microscopic studies have shown that about 70% of spines contain the PSD protein drebrin under normal conditions that regulate spinogenesis and synaptogenesis. Specifically, drebrin A appears to be implicated in the arrangement of the dendritic actin pool for the composition of spines and excitatory axospinous synapses during the early postnatal periods [390]. However, competitive blockades of NMDAR induce rapid changes in the distribution of F-actin and drebrin A within dendritic spines, suggesting their glutamate-dependent modulation [36,391]. Furthermore, clinical studies have reported that the levels of drebrin A are significantly reduced in the brain of patients with major cognitive impairment, such as Alzheimer’s disease [392] and schizophrenia [381]. Interestingly, post-mortem studies have reported decreased drebrin levels in the temporal cortex and increased levels in the frontal cortex of patients with mild cognitive impairment, suggesting a disparity in postsynaptic efficacy in these regions during the onset of Alzheimer’s disease. This differential expression of drebrin in the temporal and frontal cortex in Mild Cognitive Impairment patients suggests progressive cognitive dysfunction, associated with initial deterioration of associative behaviors such as memory and language, and relative preservation of executive functions during the early stages of cognitive decline [392].

### 9.3. Spinophilin Regulation in the Dynamic Morphological Changes of Dendritic Spine: Implications in Schizophrenia

Several pieces of evidence have emphasized the role of the PSD protein spinophilin, which is responsible for interacting with several highly enriched proteins in spines [393,394], such as actin [394] and protein phosphatase-1 (PP-1) [394,395]. Several in vitro studies have found that spinophilin is capable of binding actin filaments [393], suggestive of a role as an actin-based cytoskeleton organizer in dendritic spines. Although PP-1 has been shown to modulate the activity of ion channels, including NMDAR [396], this function resulted in the absence of spinophilin. Its role in regulating excitatory glutamatergic transmission by anchoring PP-1 to ionotropic glutamate receptors (Figure 2). Preclinical studies using spinophilin KO mice have proven that spinophilin modulates both glutamatergic synaptic transmission and dendritic morphology. Specifically, the ability of PP-1 to regulate the activity of NMDARs was found to be reduced in spinophilin KO mice. Additionally, spinophilin gene deletion reported evidence of increased spine density during development and altered filopodial formation in neurons, consistent with its function as a regulator of the dendritic spine [397]. The mechanism by which spinophilin might regulate dynamic morphological changes in the dendritic spine could be an interaction with the actin cytoskeleton [393], which is known to influence spine morphology [398]. In particular, spinophilin can bind F-actin filaments [393] and within the binding domain has several phosphorylation sites for PKA, PKC, and CaMK, which are responsible for regulating cytoskeletal reorganization (Figure 2). Furthermore, PP-1 has been shown to modulate cell morphology through de-phosphorylation of the actin cytoskeleton [399]. These findings suggest that direct interactions of spinophilin with actin and PP-1 may provide a highly localized mechanism for regulating the phosphorylation state of the actin cytoskeleton. In this context, the modulation of glutamate receptor activity by spinophilin provides another possible effect on spine density as the enhancement of NMDAR transmission by PP-1 inhibitors observed in wild-type mice that was significantly attenuated in KO mice, suggesting that modulation of NMDARs by PP-1 was lost in spinophilin-deficient neurons. Dysregulation of glutamate receptor pathways in spinophilin-deficient neurons appears to lead to specific changes in neuronal circuits [397]. In addition to NMDARs, spinophilin can directly interact with D2Rs implicated in the pathophysiology of schizophrenia and targeted by antipsychotics, suggesting an implication of spinophilin in schizophrenia [394,400] (Figure 3). It has been hypothesized that spinophilin has a role in establishing a signaling complex for dopaminergic neurotransmission through D2Rs, linking receptors to downstream signaling molecules and the actin cytoskeleton [393,400]. Post-mortem studies determined the spinophilin expression in synaptosomal membranes (SPM) and PSD fractions in the DLPFC of schizophrenia patients without antipsychotics vs. antipsychotic-treated and HC. The results of this study showed that spinophilin was significantly reduced in patients with schizophrenia compared with HC in both SPM and PSD fractions (*p* = 0.007 and *p* = 0.039, respectively). Moreover, spinophilin was significantly reduced in patients treated with antipsychotics (*p* = 0.003 for SPM and *p* = 0.014 for PSD), but not in those without antipsychotics, suggesting that the alterations in the expression of spinophilin potentially might be implicated in the side effects of this class of compound [149].

## 10. Discussion and Future Directions

The results reported in this systematic review are discussed here covering three main aspects. Firstly, the main implications that have emerged for PSD proteins with the most robust experimental and clinical data in schizophrenia. Secondly, the role of PSD proteins in the framework of dopamine–glutamate interactions. Thirdly, the translational value of PSD proteins in light of potential innovative therapeutic targets. Multiple lines of evidence support the concept that changes in PSD proteins may be associated with schizophrenia, consistent with the idea of this condition as a synaptopathy [401]. This evidence comes from various “omics” strategies, including GWAS of patients [402], post-mortem proteomics and transcriptomics studies [403], as well as in vivo, ex vivo [404], and in vitro [405] models. However, in silico bioinformatics experiments [406] and machine learning applications [407] have contributed in the last year to strengthen the role of PSD proteins in schizophrenia-related synaptopathy [408]. Finally, the possibility that such alterations at the micro- and nano-domain level of the synapse may, acting in synchrony and synergy, extend their consequences to a higher scale of brain function is beginning to be explored by connectivity and connectome analysis [401,409]. Advances in PSDs have unveiled a kind of “molecular Lego” that assembles in the glutamate synapse a sophisticated network in which a single PSD protein (e.g., Shank3 or Homer1) can cooperate on specific signaling or can cooperatively converge by interacting with other PSD proteins on the same signal transduction pathway [8]. This interaction is highly self-organized and occurs rapidly, at least as demonstrated by reconstitution experiments in which PSD proteins exchanged rapidly between condensed fluorescence recovery after photobleaching analyses [410]. The involvement of the Shank protein family, and in particular Shank3, in the genetics of schizophrenia as well as ASD has greatly strengthened the notion that the two classes of disorders, albeit with different ages of onset, clinical trajectory, and diagnostic criteria, may share a genetic background at least for a subgroup of patients. It has confirmed what is already known about the genetics of major psychiatric disorders (bipolar disorder, major depression, and schizophrenia) that share genes particularly involved in immune function, epigenetic regulation, and PSD function. Homer proteins, in particular Homer1 b/c and its early gene isoforms, such as the short Homer1a, act as dominant negatives, have been found to be altered in the post-mortem brain of schizophrenic patients [11] and implicated in modeling the dopamine-glutamate pathophysiology of the preclinical disease proxy [8]. Homer has been shown to play a central role in the construction and modeling (together with PSD-95) of the dendritic spine [50,411,412] and recent evidence has indicated Ankyrin-G as a topologically important crucial node recognized by the EVH1 domain [277]. A more conservative approach to understanding Homer1 in schizophrenia should also take into account the fact that this gene and its transcripts have been associated with broader physiological implications, such as higher cognitive and executive functions, as demonstrated by the effect of genetic variants in modulating working memory [267,268]. Furthermore, DISC1 and Neuregulin are among the PSD proteins with probably the most genetic data available and have been included among the genes contributing to disease susceptibility [10,187]. Considering the peculiar role of these molecules in tuning synaptic function by bridging pre- and postsynaptic structure and function, both molecules extend the role of PSD in disease by highlighting the structural and functional overlapping between pre- and postsynapses [413]. Furthermore, the intersection of Shank3 with the pathophysiology of schizophrenia has recently been marked by the discovery of several point mutations and small deletions of *Shank3* [61], as well as the de novo R1117X mutation leading to loss of function of the Shank3 protein causally associated with schizophrenia [64]. Finally, a translational feature directly related to the pharmacotherapy of schizophrenia is the robust effect emerging from animal models of antipsychotic treatment is the differential gene expression at the PSD. This has been demonstrated by antipsychotics alone or in co-administration with other psychotropic drugs, such as mood stabilizers, with and without pretreatment with drugs that mimic the pathophysiology of psychotic behavior [104,116,337]. Critical appraisal of the role of PSD in the pathophysiology of schizophrenia cannot be limited to the evidence and the role, albeit relevant, of proteins that have been firmly implicated in the illness without attempting to place PSD within the overall framework of dopamine-glutamate interaction. In this respect, this interplay appears to be one of the most robust and explored in schizophrenia, although not the only one. It would be conceivable to hypothesize a bidirectional mechanism [414] with dopamine perturbation influencing PSD expression, such as for *Homer* [415]. Recently, a comprehensive post-mortem analysis was performed in the caudate nucleus of 245 HCs, 154 patients with schizophrenia and 44 patients with bipolar disorder from different countries (210 Africans and 233 Europeans) with an analysis of transancestral quantitative expression loci (eQTLs) and hundreds of specific cis-eQTLs. The integration of quantitative expression loci analysis, Mendelian randomization with the most recent GWAS association study on schizophrenia, the transcriptome-wide association study, and differential expression analysis allowed the identification of many genes associated with schizophrenia risk, potentially including the short isoform of the dopamine D2R. It is plausible to hypothesize that antipsychotic drugs have an influence on caudate gene expression and that their networks highlighted interactions implicated in schizophrenia risk providing a resource for potential future therapeutic targets [416]. A new and unconventional way of considering the effect of dopamine on the glutamatergic synapse and possibly influencing PSD function derives from the Pajet–Blanc experiment that demonstrated the rapid release of dopamine at striatal varicosities and its effect on the glutamatergic synapse. In this context, the adhesion of dopaminergic synapses to glutamatergic, GABAergic, or cholinergic synapses is organized into a “dopamine hub synapse” supporting local dopaminergic signaling and integrating the volumetric transmission crucial for the modulation of monoamines network activity [417]. Therefore, we could evaluate the current position of PSD in schizophrenia by considering its strengths and weaknesses. Among the strengths, two stand out. The first is the genetic background derived from GWAS or SNPs and the discovery of rare variants that recapitulate several phenotypic aspects of schizophrenia or the syndromic presentation of schizophrenia. The second is the translational value of the preclinical in vivo and ex vivo findings that, by specifying the discrete molecular mechanisms of PSD-mediated synapse function (i.e., growth and formation of dendritic spines), have highlighted the potential molecular mechanisms responsible for the complex behavior and their abnormal expression. Weaknesses include the difficulty in assigning to each protein interacting in a large network (e.g., Homer-PSD95-Shank) with other molecules a specific weight in contributing to a complex disorder such as schizophrenia.

In conclusion, currently, PSD proteins at the glutamatergic synapse level have emerged as one of the major constituents of the dendritic spine implicated in the molecular pathophysiology of schizophrenia, corroborating the concept that this disorder may be inherent at the cellular level as a synaptopathy. Abnormal expression of synaptic function at the micro-macrodomain and higher levels may influence multiple aspects of the pathophysiology of schizophrenia. In this regard, PSD proteins may influence the spatial and temporal organization of dendritic spines, thus are significant players in the complex intersection of neurodevelopment and context-related factors implicated in the pathophysiology of schizophrenia, its clinical phenotypes, and possibly treatment effects.

## Figures and Tables

**Table 1:** PSD proteins expression and signaling. Arc = activity-regulated cytoskeleton-associated protein; SR = serine racemase; SR^−/−^ = serine racemase knockout; WT = wild-type; KO = knock-out; LRRC7 = leucine rich repeat containing 7; NMDAR = N-methyl-D-aspartate receptor; DISC1= disrupted-in-schizophrenia 1; SDS-PAGE = sodium dodecyl sulphate-polyacrylamide gel electrophoresis; CaMKII = calcium/calmodulin-dependent protein kinase II; SCZ = schizophrenia; ASD = autism spectrum disorders; mGluR5 = metabotropic glutamate receptor 5; HPLC = high performance liquid chromatography; Ro61-8048 = 3,4-dimethoxy-N-[4-(3-nitrophenyl)thiazol-2-yl]-benzene-sulphonamide; GluN2A = glutamate ionotropic receptor NMDA type subunit 2A; GluN2B = glutamate ionotropic receptor NMDA type subunit 2B; DBZ = DISC1-binding zinc finger protein; PSD = postsynaptic density; UHVEM = Ultra-High voltage electron microscopy; TEM = transmission electron microscopy; FRET = immunofluorescence fluorescence resonance energy transfer; NPY = neuropeptide Y; D2R = dopamine D2 receptor; GSK3β = glycogen synthase kinase 3 beta; TAT = Trans-activator of transcription; STORM = stochastic optical reconstruction microscopy; TG = transgenic; mPFC = medial prefrontal cortex; GPR85 = G Protein-Coupled Receptor 85; LTP = long-term potentiation; HEK 293 = human embryonic 293; shRNA = single hairpin; SAP= synapse-associated protein; ERK = extracellular signal-regulated kinase; qPCR = quantitative real-time PCR; SAP97-cKO = brain-specific conditional knockout of SAP97; MAGUKs = membrane associated guanylate kinases; DLGAPs = disk large associated guanylate associated proteins; NMR = nuclear magnetic resonance; SH3 = src homology 3; Cav1.3 = calcium channel, voltage-dependent, L type, alpha 1D subunit; Slitrk2 = SLIT and NTRK-like family, member 2; ErbB2 = erb-b2 receptor tyrosine kinase 2; ErbB4 = erb-b2 receptor tyrosine kinase 4; GABA = gamma-aminobutyric acid; TACE = TNF-alpha-converting enzyme; GlyT1^+/−^ = glycine transporter subtype 1 heterozygote mutant; HT = heterozygous; SULT4A1 = Sulfotransferase 4A1; BiFC = bimolecular fluorescence complementation; AI = ibotenic acid; sIPSCs = spontaneous inhibitory postsynaptic currents; HB-EGF = Heparin-binding epidermal growth factor-like growth factor; ↓ = decrease; ↑ = increase; ELISA = enzyme-linked immuno-sorbent assay; NLGN = neuregulin; mEPSCs. = miniature excitatory postsynaptic currents; CRFR1 = Corticotropin-releasing factor receptor 1; AMPA = α-amino-3-hydroxy-5-methyl-4-isoxazolepropionic acid; BDNF = brain-derived neurotrophic factor.

PSD Protein	Study Design	Methodology/ Samples	Species	Signaling	Functional Outcome	Authors
Arc	In vivo preclinical study	Golgi staining Western blot analysis	Mice (WT and SR^−/−^)	SR is the enzyme that converts L-serine to D- serine, a coagonist of NMDAR. SR^−/−^ mice ↓ Arc in the hippocampus.	D-serine chronic treatment reverses the Arc expression alterations and rescues the hippocampal spine deficit in SR^−/−^ mice.	Balu et al., 2014 [70]
DISC1	Preclinical study	Immunoblot analysis Immunocytochemistry SDS-PAGE	Mice (KO, exon 3 deletion in LRRC7)	Densin, encoded by LRRC7, produces a high-affinity complex with α-CaMKII and α-actinin. Deletion of densin-180 ↓ mGluR5 and DISC1 levels.	Signaling defects by loss of densin alter the morphology of the spine and even produce behavioral endophenotypes that are SCZ- or ASD-like,	Carlisle et al., 2011 [71]
	Preclinical study	Electrophysiology Immunoblotting HPLC	Rats (prenatal)	Ro61-8048 ↓ GluN2A and ↑GluN2B in embryos’ brains induce changes in sonic hedgehog at 24 h and ↑ neuronal excitability of coupled impulses. DISC1 was not modified.	The tryptophan–kynurenine pathway, influencing NMDAR, leads to synaptic changes in the embryonic and neonatal brain.	Forrest et al., 2013 [72]
	Preclinical study	UHVEM, TEM	Mice (DBZ KO)	In DBZ KO mice, ↓ dendritic arborization, ↑ density of spines (especially thin spines), and ↓ PSD number in the spines.	DBZ deficiency ↑ dendritic spine of cortical pyramidal neurons.	Koyama et al., 2015 [73]
	In vitro preclinical study	FRET, STORM Western blot Analysis	Mice	D2R and DISC1 complexes form clusters in the dendritic spines of striatal neurons. TAT-D2pep, assisted by NPY and GSK3β, ↓ D2R-DISC1 complexes, and even ↑ dendritic spines, synaptophysin, and PSD-95.	TAT-D2pep has a protective effect on neuronal morphology and dendritic spines.	Zheng et al., 2020 [74]
Neuregulin	Preclinical study	Co-immunoprecipitation	Mice (erbB2/B4 KO and WT)	NRG1/erbB4 promotes dendrite growth in mature interneurons through kalirin. Phosphorylation in the C-terminal of kalirin-7 is important for the reduction in intraneuronal dendrite length.	The neural effects of kalirin (modulation of NRG1/erbB4 and dendrite growth) seem to be correlated with the SCZ onset.	Cahill et al., 2012 [75]
	In vitro preclinical study	Immunostaining Immunoblot	Human lymphocytes (72 ASD patients)	NLGN at the level of PDZ3 interacted with PSD-95. Two patients with ASD had inherited missense mutations in conserved sites (T1033C/M152T and G1239T/V221L) of GPR85 related to G-protein interaction and signal transduction. Mutated GPR85 was preferentially accumulated, causing endoplasmic reticulum stress, and disrupting dendrite formation of hippocampal neurons, whereas, in GPR85 WT, this did not occur.	Mutated GPR85 in ASD patients disrupted dendrite formation through disconnection between GPR85 and NLGN via PSD-95, which is potentially implicated in ASD.	Fujita-Jimbo et al., 2015 [76]
	In vitro preclinical study	Immunocytochemistry Western Blot	Mice (ErbB4^−/−^ and ErbB4^+/+^)	There was no difference in the size of the inhibitory synapse Gephyrin clusters in the dendrites of GABA-ergic cortical neurons between ErbB4^−/−^ and ErbB4^+/+^ mice. The number of neuronal dendrites and Gephyrin inhibitory postsynaptic proteins in the ErbB4^−/−^ group were lower. ErbB4^−/−^ affected excitatory synapses in intermediate neuronal cells and inhibitory synapses in pyramidal neurons but did not result in changes in inhibitory ones.	The ErbB4 gene has no effect on either inhibitory synapses in interneurons or excitatory synapses in pyramidal neurons. NRG1 and ErbB4 signaling are necessary for the postnatal cortical GABA network.	Li et al., 2020 [77]
	Preclinical study	Semi-quantitativeRT-PCR Immunofluorescence	Mice	Neuronal ErbB-4 is most prevalent in GABAergic interneurons. The fraction of ErbB-4 receptors on the plasma membrane ↑ from 30% to 65% between 6 and 16 days in vitro; from stimulation by NRG, PSD-95 also ↑. In addition, TACE in hippocampal cultures is resistant to cleavage, especially the JM-b isoform.	At glutamatergic synapses, signaling is influenced by ErbB-4.	Logart et al., 2007 [78]
	Preclinical study	Western Blot Immunoprecipitation	Rats	After one day of being treated with MK-801, the rats exhibited ↑associations of ErbB4 with PSD-95 and NMDAR in the PFC, while only phosphorylated ErbB4 relative to ErbB4 was ↑in the hippocampal area, although these changes were detectable at 12 days. Moreover, such treatment led to a reduction in locomotor capacity and the onset of an anxiety-like phenotype.	Repeated blockade of NMDAR could modulate NRG1-ErbB4 signaling.	Li et al., 2013 [79]
	Preclinical study	Immunostaining Western Blot Electrophysiological	Mice	NRG1 ↑ PSD-95 and mEPSCs in GABAergic interneurons, while it had no effects on excitatory synapses in glutamatergic neurons. In addition, the deletion of ErbB4 in parvalbumin-positive interneurons results in reduced mEPSCs.	ErbB4 has a pivotal role in the synaptogenesis of excitatory synapses, while NRG1 has a specific effect on synapses of GABAergic interneurons.	Ting et al., 2011 [80]
PSD-95	Preclinical study	Western blot analysis	Mice (GlyT1^+/−^ and SR^−/−^)	SR^−/−^ mice had elevated NR1 and NR2A NMDAR protein levels in the hippocampus but not in PFC as in GlyT1^+/−^ mice. In contrast, there were no changes for AMPAR (GluR1, GluR2) or PSD95 in either region. GlyT1^+/−^ mice had high expression of NR1, NR2A, GluR1 and GluR2 in PSD.	Genetic perturbation and ionotropic glutamate receptor alterations in the PSD of hippocampal neurons are implicated in SCZ.	Balu et al., 2011 [81]
	*Post-mortem* study	Immunoprecipitation Western blot analysis	Mice and human brain tissue	NMDA receptor complexes in the PSD are ↑ in the SCZ. The DLPFC of SCZ cases shows an ↑ in PSD-95 and erbB4 and an ↓ in RPTPa and dysbindin-1, each of which reduces Src activity through protein interaction with Src.	In SCZ, there is a reduction in NMDA receptor signaling at the post-receptor level, and Src is probably responsible for these dysregulations.	Banerjee et al., 2015 [82]
	Preclinical study	Immunoprecipitation Western blot analysis	Mice (Dlgap1 WT, HT, and KO)	DLGAP1 protein is localized in glutamatergic neurons and is a component of protein complexes organized by PSD95.	Dlgap1 KO mice had sociability deficits and disruption of protein interactions in PSD.	Coba et al., 2018 [83]
	Preclinical study	Co-immunoprecipitation Western blot	Mice (PSD-95^−/−^)	During adolescence in the mice analyzed, there was an ↑ in total protein levels of the NMDAR subunits GluN1 and GluN2B, with an ↓ in the AMPAR subunit GluA1.	PSD-95^−/−^ mice showed a lack of sociability and deficits in learning and working memory with altered mPFC synaptic function.	Coley et al., 2019 [84]
	Preclinical study	Immunocytochemistry Immunoprecipitation Electrophysiology	Mice	SULT4A1 modulates neuronal branching and dendritic spine formation; also, by negatively regulating the catalytic activity of Pin1 for PSD-95, it facilitates the expression and synaptic function of NMDAR.	Inhibition of Pin1 reverses the pathological phenotypes restoring dendritic spine density and improving NMDAR-mediated synaptic transmission.	Culotta et al., 2020 [85]
	Preclinical study	Immunoprecipitation Mass spectrometry	Mice (PSD-95^TAP/+^)	Homozygous mice showed no alterations in PSD-95 expression, subcellular localization, and synaptic function.	PSD-95 is a SCZ susceptibility protein.	Fernandez et al., 2009 [86]
	Preclinical study	Electrophysiology Immunoblotting	Rats	Ro61-8048 administration determined sonic hedgehog expression ↑, as did levels of doublecortin and PCNA, suggesting activation of neurogenesis. Moreover, the PSD-95 ↑.	The kynurenine pathway determined functional synaptic changes in the embryonic and neonatal nervous system influencing the expression of proteins that modulate NMDAR.	Forrest et al., 2013 [72]
	In vivo preclinical study	Co-immunoprecipitation Immunocytochemistry Immunohistochemistry Electrophysiology	Mice (SNAP-25 WT and HT)	SNAP-25 includes PSD-95 and p140Cap. Acute downregulation of SNAP-25 in CA1 affects the number of dendritic spines, and hippocampal neurons from mice heterozygous for SNAP-25 show reduced densities of spines and alterations in PSD-95.	SNAP-25 is important in controlling PSD-95 and may contribute to SCZ through an effect on postsynaptic function and plasticity.	Fossati et al., 2015 [87]
	*In vitro* preclinical study	Immunostaining Immunoblot	Human lymphocytes (72 ASD patients)	The C-terminal sequence of GPR85 at the level of PDZ1 interacted with PSD-95, while NLGN at the level of PDZ3 interacted with PSD-95.	GPR85 carrying the mutations disturbed dendrite formation that could be implicated in the pathogenesis of ASD through the associated NLGN-PSD-95 receptor complex.	Fujita-Jimbo et al., 2015 [76]
	Preclinical study	Co-immunoprecipitation Immunohistochemistry Western Blot	Rats	The expression of PSD-95 ↑ in the medial PFC of maternal separation-exposed adolescents, while NR2A did not significantly ↑. This effect resulted in behavioral alterations.	The ↑ of NR2A in PFC is related to SCZ and acts largely through its association with the postsynaptic protein PSD-95.	Ganguly et al., 2015 [88]
	Preclinical study	BiFC Co-immunoprecipitation Immunoblotting Immunocytochemistry	Rats	Calcyon interacting with PSD-95 is then able to form a ternary complex with D1DR. Calcyon when phosphorylated ↑ the association with PSD-95.	Calcyon modulates trafficking of D1DR by forming a ternary complex with it via PSD-95.	Ha et al., 2012 [89]
	Preclinical study	RT-PCR cDNA Constructs	Mice (Shank3- overexpressing TG)	The expression of Gpr85 was ↑ in multiple brain regions of the mice, while the mRNA levels of Gpr27 and Gpr173 ↓.	Gpr85 was colocalized with PSD-95 and Shank3 mice brain.	Jin et al., 2018 [90]
	Preclinical study	Immunoprecipitation Western bot	Rats	One day after MK-801 administration in rats, the associations of ErbB4 with PSD-95 and NMDAR were ↑ in the PFC, whereas in the hippocampus only phosphorylated ErbB4 ↑. These effects tended to fade chronically, furthermore, long-term treatment resulted in reduced locomotor capacity and the onset of an anxiety-like phenotype.	NRG1-ErbB4 signaling could be modulated by repeated blockade of NMDAR with PSD-95.	Li et al., 2013 [79]
	In vitro preclinical study	Immunocytochemistry Western blot	Mice (ErbB4−^/^− and ErbB4^+/+^)	PSD-95 on the neuronal surface was significantly lower than in 1NMPP1, while Gephyrin on the neuronal surface was equally present.	The density of PSD-95 in GABAergic neurons was not different in the controls.	Li et al., 2020 [77]
	In vivo preclinical study	Immunocytochemistry	Mice	Slitrk2 directly interacts with PSD-95 through a SH3 domain binding motif. Furthermore, PSD-95 induces robust clustering of Slitrk2 in 293T cells. The deletion of the SH3 domain in PSD-95 or the SH3 domain binding motif in Slitrk2 reduces this clustering.	PSD-93 and PSD-95 can form molecular complexes with NRG and K+ channel and are associate with Slitrk2 in postnatal mouse brain.	Loomis et al., 2020 [91]
	Preclinical study	Immunocytochemistry Immunoprecipitation Western bot	Mice	SR binds both PSD-95 and stargazin, this results in inhibition of the SR enzyme. This complex is blocked, triggering the synthesis of D-serine, by activation of AMPA receptors.	The interactions of SR-stargazin-PSD-95 modulated NMDA/AMPA receptor.	Ma et al., 2014 [92]
	Preclinical study	Western bot ELISA	Rats	The density of dendritic spines and their content of PSD-95, Syn, AMPA receptors, and BDNF ↓ in AI-injured rats. In addition, neural architecture was changed with thin, mushroom-shaped, stubby and broad spines.	In AI-injured animals, working memory and social behavior were altered, as well as PSD-95 and AMPA receptors.	Martinez-Torres et al., 2021 [93]
	Preclinical study	Co-immunoprecipitation Electrophysiology Western bot	Mice (PSD-95+/−)	In HT mice, there was an ↑ in α1 subunit of the GABAA receptor and an ↑ in sIPSCs in mPFC pyramidal neurons, with a significant ↑ in evoked IPSCs, leading to excitatory–inhibitory dysregulation in such mice. PSD-95 deficiency enhances the functioning of inhibitory synapses through upregulation and trafficking of NLGN2 and reduction of GSK3β activity.	PSD-95 deficiency results in effects on GABAergic transmission in mPFC.	McEachern et al., 2020 [94]
	Preclinical study		Mice (HB-EGF KO)	KO mice had behavioral abnormalities ameliorable by antipsychotic treatment, altered dopamine and serotonin levels, ↓ spines in PFC neurons, ↓ CaMK II, NR1 and PSD-95.	Alterations in HB-EGF signaling may predispose SCZ, through dysregulation, of many factors, including PSD-95.	Oyagi et al., 2009 [95]
SAP97	Preclinical study	Western Blot	Mice	CRFR1 acts with SAP97, HEK 293, and cortical brain lysates. This interaction depends on an intact PDZ binding motif at the carboxyl-terminal tail end of CRFR1. SAP97 is recruited to the plasma membrane in HEK 293 cells expressing CRFR1, and mutation of the PDZ-binding motif of CRFR1 results in the redistribution of SAP97 in the cytoplasm. SAP97 overexpression inhibits internalization of CRFR1. Knockdown with shRNA of SAP97 did not affect cAMP, but knockdown of SAP97 attenuated CRFR1-stimulated phosphorylation of ERK1/2.	SAP97 interactions with CRFR1 attenuate CRFR1 endocytosis and SAP97 is involved in the coupling of G-protein-coupled receptors to ERK1/2 signaling pathway activation.	Dunn et al., 2013 [96]
	Preclinical study	qPCR Western Blot RNA sequencing	Mice (SAP97-cKO)	RNA sequencing of hippocampi from male control and SAP97-cKO animals identified 67 transcripts regulated by SAP97. Male-specific cognitive impairment and female-specific motor impairment were found in SAP97-cKO mice, while other behaviors remained essentially unchanged.	Loss of SAP97 is associated with SCZ, and it may have a contribution in the behavior.	Gupta et al., 2018 [97]
Shank	Preclinical study	Immunocytochemistry Immunohistochemistry RT-PCR Western Blot MicroArray Mass-spectrometry	Mice	De novo mutation R1117X in ProSAP2/Shank3 leads to its accumulation in the nucleus. This alters the transcription of some genes involved in SCZ, such as Synaptotagmin 1 and LRRTM1. In addition, hippocampal neurons with the ProSAP2/Shank3 SCZ mutation show altered ratio and reduced dendritic branching.	The uncoupling of nuclear shuttling of ProSAP2/Shank3 from synaptic activity may represent one of the mechanisms underlying the pathogenesis of SCZ.	Grabrucker et al., 2014 [61]
	Preclinical study	Chromatography NMR spectroscopy	Mammalian cells	Mammalian Shank proteins have identical and typical SH3 folding motifs but unusual target binding pockets.	There is an atypical interaction where SH3 domains binds to a region of Cav1.3.	Ishida et al., 2018 [98]
	Preclinical study	RT-PCR cDNA Constructs	Mice (Shank3- overexpressing TG)	In Shank3 TG mice several myelin-related genes were downregulated specifically in mPFC, but not in the striatum or hippocampus. mRNA levels of Gpr85 ↑ in multiple brain regions of the mice brain, while mRNA levels of Gpr27 and Gpr173 ↓ in cortex and striatum.	Molecular changes of overexpressing Shank3 in mice brains could be implicated in the pathogenesis of SCZ.	Jin et al., 2018 [90]
	Preclinical study	Liquid chromatography–tandem MS	Mice	LTP results in the reorganization of PSD, which in turn links glutamate receptor signaling to kinases and phosphatases as well as target proteins modulated by protein phosphorylation.	Phosphoproteins regulated by LTP, including the scaffold proteins Shank3, Syngap1, Dlgap1 and Dlg4, were a risk factor for SCZ and autism spectrum disorder.	Li et al., 2016 [99]
	Preclinical study	Biochemistry Mass spectroscopy	Mice	There is a central PSD scaffold at age 14, but while the NMDAR subunits are already linked to MAGUKs and DLGAPs protein complexes, the downstream components are held in separate protein complexes organized by SHANKs. There is a continuous expansion of functional groups and ↑ associations to glutamate receptors in the upper layers and proteins with embedded enzyme activities in the middle and lower scaffold layers. The scaffold interactors form a more heterogeneous set of protein complexes than the basic PSD scaffolds.	The network of protein interactions of the adult PSD is connected through a core scaffold component that uses specific hubs for localization. Instead, a number of PSD scaffold interactors may also associate in protein networks enriched in “housekeeping” protein interactions early in development that are not exclusive to neurons.	Li et al., 2017 [100]
	Preclinical study	Chromatography	Rats	The binding specificities of PDZ domains of different Shank are similar. The structure in solution of Shank3 in tandem containing the SH3 and PDZ domains showed that the two domains are close to each other. The SH3 domain did not affect the affinity of the PDZ domain toward short target peptides.	The R536W mutation of Shank3 (implicated in SCZ) in the binding between the domains had no effect on the structure or peptide interactions of the Shank3 SH3-PDZ unit.	Ponna et al., 2018 [101]

**Table 2:** PSD proteins expression after drug administration in preclinical or post-mortem study. Arc = activity-regulated cytoskeleton-associated protein; ASE = asenapine; OLA = olanzapine; HAL = haloperidol; MIN = minocycline; SER = sertindole; KET = ketamine; AMS = amisulpride; ACC: anterior cingulate cortex; MAC = medial agranular cortex; SS = somatosensory cortex; IC = insular cortex; NAc = nucleus accumbens; M1 = motor cortex; M2 = medial agranular cortex; dlCP = dorsolateral caudate–putamen; dmCP = dorsomedial caudate-putamen; vmCP = ventromedial caudate-putamen; vlCP = ventrolateral caudate–putamen; DISC1 = disrupted-in-schizophrenia 1; NLGN = neuroligin; SHANK = SH3 and multiple ankyrin repeat domains; PSD = postsynaptic density; DLGAP = disks large-associated protein; VPA = valproate; AT = antipsychotic; PFC = prefrontal cortex; PrL = prelimbic cortex; VO = ventral orbital cortex; LO = lateral orbital cortex; DLO = dorsolateral orbital cortex; Ddo^−/−^ = D-aspartate-oxidase knockout mice; DAT = dopamine transporter; VTA = ventro-tegmental area; SNc = substantia nigra pars compacta; SCZ = schizophrenia; CAMKII = calcium/calmodulin-dependent protein kinase II; TER = terguride; CLO = clozapine; ZIP = ziprasidone; MEM = memantine; NRG = neuregulin; ErbB4 = Erb-B2 Receptor Tyrosine Kinase 4; ↓ = decrease; ↑ = increase; PN = postnatal days; PCP = phencyclidine;; p-ErbB4 = phosphorylated-Erb-B2 receptor tyrosine kinase 4; DLG = discs large MAGUK scaffold protein; NMDA = N-methyl-D-aspartate; pS295 = A phosphorylated state of PSD-95; JNK = Jun N-terminal kinase; DLPFC = dorsolateral prefrontal cortex; Cdk5 = cyclin dependent kinase 5; Rack1 = receptor for activated C kinase 1; NR2 = human NMDA receptor 2; KD = knockdown; SNP = single nucleotide polymorphism; ASD = autism spectrum disorder; MDD = major depressive disorder; VGLUT1 = presynaptic vesicular glutamate transporter 1; APZ = aripiprazole; LTP = long-term potentiation; Akt = serine/threonine kinase family; GSK3 = glycogen synthase kinase-3; PCC = posterior cingulate cortex; BD = bipolar disorder; NF-L = neurofilament light chain protein; DG = dentate gyrus; Sub = subiculum; PDZ = postsynaptic density-95/discs large/zone occludens-1; PMDS = Phelan–McDermid Syndrome; CME = clathrin-mediated endocytosis; qRT-PCR = real-time quantitative reverse transcription polymerase chain reaction; ECL = chemiluminescence; BDNF = brain-derived neurotrophic factor.

PSD Protein	Study Design	Methodology/ Samples	Species	Drug Intervention	Functional Outcome	Authors
Arc	Preclinical study	In situ hybridization	Rats	HAL (In acute)	HAL ↑Arc in NAc, dlCP, dmCP, vmCP and vlCP. MIN ↓Arc in the same regions. Moreover, HAL add-on to MIN ↓ Arc in ACC, MAC, M1, SS, and IC.	Buonaguro et al., 2017 [106]
	Preclinical study	In situ hybridization, Probe radiolabeling	Rats	ASE, HAL, OLA (In acute)	HAL ↑ Arc in NAc (core and shell), dmCP, dlCP, and vlCP; and HAL ↓ Arc in ACC, M2, SS, and IC. ASE ↑ Arc in NAc core, dlCP, and vlCP. ASE or OLA ↓ Arc in ACC, M1, M2, dlCP, vlCP, and NAc core.	de Bartolomeis et al., 2015 [107]
	Preclinical study	In situ hybridization, Western blot analysis	Rats	HAL (In acute)	HAL ↑ Arc expression in dlCP, dmCP, and vlCP and ↓ Arc in M2, SS, and IC.	de Bartolomeis et al., 2018 [108]
	Preclinical study	In situ hybridization, Probe radiolabeling	Rats	HAL, SER (In acute)	Arc was ↑ by HAL in dlCP, dmCP, vmCP, vlCP, and NAc, (only core). SER has no significant effects on Arc expression.	Iasevoli et al., 2010 [109]
DISC1	*Post-mortem* study	Immunohistochemistry, Immunolabeling, Western blot analysis	Human (7 normal brain tissue)	-	The presence of DISC1 in multiple types of frontal and parietal cortex (BA 4, 9, 39, and 46) synapses, in ribosomes, dendritic trees, microtubules, Golgi apparatus, and multivesicular bodies suggests that DISC1 may participate in synaptic activity.	Kirkpatrick et al., 2006 [110]
Homer	In vitro preclinical study	Immunocytochemistry, Immunoblotting, Semiconductor sequencing, PCR	Human (98 patients with SCZ history and 484 controls)	-	Eighteen genes were sequenced for PSD-related proteins (NLGN, SHANK, DLGAP Homer) in SCZ patients with a psychiatric family history. In the SCZ patients’ group, a mutation of HOMER2 (c.826A>G, rs373795200) was found.	Hu et al., 2020 [10]
	*Post-mortem* study	Immunoblotting	Human (20 SCZ patients and 20 controls)	-	Alterations of the scaffold protein Homer1 (+42.9%) and Homer1b/c (− 24.6%) were evidenced in the CA1 hippocampal region in SCZ patients.	Matosin et al., 2016 [111]
	Preclinical study	In situ hybridization	Rats	HAL	HAL ↑ Homer1a in dmCP, dlCP, vmCP, and vlCP. On the contrary, Homer1b/c expression was not variated.	Buonauguro et al., 2017 [106]
	Preclinical study	In situ hybridization	Rats	HAL, ASE, OLA	HAL shifted the ratio toward Homer1b in ACC, M2, M1, and IC, while shifted the ratio toward Homer1a in the dlCP and NAc. ASE ↑ Homer1b in the IC, and SS and ↑ Homer1a in dlCP. OLA ↑ Homer1a in dlCP.	Buonauguro et al., 2017 [112]
	Preclinical study	In situ hybridization	Rats	HAL, OLA	Both AT (HAL and OLA) ↑ Homer expression in the NAc.	de Bartolomeis et al., 2002 [113]
	In vivo preclinical study	In situ hybridization, Radiolabeling, Histochemistry	Rats	Lithium, VPA (In chronic)	Lithium and VPA ↓ Homer1b/c in M1, M2, IC, dmCP, vmCP, and dlCP. Moreover, VPA ↓ Homer1b/c in NAc core.	de Bartolomeis et al., 2012 [104]
	Preclinical study	In situ hybridization	Rats	KET, MEM, MK-801	KET ↑Homer1a in IC, and ↓Homer1b in M1, dlCP, and MAC. MEM ↑Homer1b in the MAC. KET and MK-801 ↑ Homer1a/Homer1b ratio, while MEM ↓ it.	de Bartolomeis et al., 2013 [114]
	Preclinical study	In situ hybridization	Rats	ASE, HAL, OLA	OLA ↑ Homer1a in dmCP, dlCP, vlCP, vmCP, NAc shell, AC, M2, M1, and IC. HAL ↑ Homer1a in dmCP, dlCP, vlCP, vmCP, and NAc, in addition ↑ Homer1b/c in M1. ASE ↑ Homer1a in AC, M2, M1, SS, dmCP, dlCP, vlCP, vmCP, and ↑ it and Homer1b/c in NAc core. All AT ↓ Homer1b/c in AC, M2 and M1.	de Bartolomeis et al., 2015 [107]
	Preclinical study	In situ hybridization	Mice (Ddo^−/−^and Ddo^+/+^)	PCP (in acute)	Ddo^−/−^ naive mice exhibited ↓ Homer1a in the PFC, PrL, VO, LO, and DLO, ↑ Homer1b/c in the striatum. PCP ↑ Homer1a in the PFC of Ddo^−/−^ mice.	de Bartolomeis et al., 2015 [115]
	Preclinical study	In situ hybridization	Rats	AMS, HAL	AMS ↑ Homer1a in the core of NAc, while HAL ↑ it in the VL and M1. Both drugs ↑ Homer1a in dmCP and vmCP. Homer1b/c in the striatum was more ↑ by AMS compared to HAL.	de Bartolomeis et al., 2016 [116]
	Preclinical study	In situ hybridization, Western blot	Rats	HAL	Homer1a was ↑ by HAL in vmCP, dmCP, and NAc core.	de Bartolomeis et al., 2018 [108]
	Preclinical study	In situ hybridization, Histochemistry	Rats	KET	Homer1a was ↑ by KET in the vmCP and NAc. KET ↑ α-CaMKII in dlCP, vmCP, dmCP, and NAc shell, while β-CaMKII was not affected. DAT ↑ in VTA and SNc.	Iasevoli et al., 2007 [117]
	Preclinical study	Radiolabeling	Rats	HAL, TER, Others (D1/D2/D3/D4 antagonists)	Homer1a was ↑ in NAc by HAL and in vlCP by TER. In MAC and M1, it was ↑ by D1, D2 and D3 antagonists. Homer1b was ↓ by TER. In the vlCP, vmCP, and NAc shell. Furthermore, HAL ↓Homer1b in vlCP.	Iasevoli et al., 2009 [118]
	Preclinical study	In situ hybridization, Radiolabeling	Rats	HAL, SER (In acute and chronic)	In acute: HAL but not SER, ↑Homer1a in dlCP, vlCP, vmCP, and dmCP. In addition, ↓ Homer1a in ACC, MAC, and M1. Both drugs ↓ Homer1a in SS and IC. In chronic: Homer1b/c was ↑ by HAL and SER in ACC, MAC, M1, SS, and IC.	Iasevoli et al., 2010 [109]
	Preclinical study	Autoradiography, In situ hybridization	Rats	CLO, HAL, ZIP (In acute and chronic)	In acute: HAL, ZIP, and CLO ↑ Homer1a in dmCP, dlCP, vmCP, vlCP, and NAc. CLO and ZIP ↑ Homer1a in ACC, SS, IC, MAC (only CLO), and M1 (only ZIP). In chronic: HAL ↑ Homer1a in dmCP, dlCP, vmCP, vlCP, and NAc; ZIP only in dlCP, vlCP, and core of NAc. ZIP ↑ Homer1b in dlCP.	Iasevoli et al., 2011 [119]
	Preclinical study	In situ hybridization	Rats	HAL, MEM (In acute)	Homer1a was ↑ by HAL and MEM in dlCP, vlCP, vmCP, and dmCP. MEM significantly induced Homer1b/c variation in dmCP, vmCP, M1, SS, and IC.	Iasevoli et al., 2014 [120]
	Preclinical study	In situ hybridization	Rats	ASE, HAL, OLA (In acute and chronic)	HAL in acute ↑ Homer1a in dlCP, vlCP, vmCP, dmCP, and core of NAc more than OLA. No effects on Homer were found by ASE. Compared with chronic administration, acute AT administration had a greater effect on Homer1a expression.	Iasevoli et al., 2020 [121]
Neuregulin	In vitro preclinical study	Immunocytochemistry, Immunoblotting, Semiconductor sequencing, PCR	Human (98 SCZ patients with family history, 484 nonrelated with SCZ, 533 controls)	-	PSD-related genes, such as NLGN, SHANK, and DLGAP, have rare functional mutations (e.g., NLGN3 c.584G>A p.R195Q rs190164205) that could be related to pathogenesis of SCZ.	Hu et al., 2020 [10]
	*Post-mortem* study	Immunoprecipitation, Immunoblotting	Human brain tissue (14 SCZ patients, 14 controls	-	NRG1 and erbB4 levels in PFC did not differ between groups. SCZ patients showed ↑ erbB4-PSD-95 interactions. In addition, stimulation of NRG1 downregulates NMDA receptor activation in the PFC.	Hahn et al., 2006 [122]
	*Post-mortem* study	Immunoprecipitatiom, Immunoblotting	Human PFC (14 SCZ patients, 14 controls)	-	NMDA in SCZ patients appeared sensitive to the NRG1inhibition, that mediates the stimulation of ErbB4. The Erb-PSD-95-NMDA complex is implicated in SCZ.	Morrison et al., 2007 [123]
	Preclinical study	ECL, Western blot	Rats (Perinatal model)	PCP	PCP treatment in the cortex determined ↓NRG1 and ErbB4 at PN12 and ↑in ErbB4 and p-ErbB4 at 5 weeks, and ↓ErbB4 and p-ErbB4 together with an ↑Nrg1 at 20 weeks. In contrast, in the hippocampus, only determined ↓ p-ErbB4 at 20 weeks.	Du Bois et al., 2012 [124]
PSD-95	*Post-mortem* study	Real-time quantitative, RT-PCR	Human brain tissue (1539 SCZ patients, 2012 controls)	-	PSD95 group (DLG 1-4) is the most important postsynaptic density component in glutamatergic synapses. SCZ patients’ haplotypes of DLG4, rs2242449, rs17203281, rs390200, rs222853, and rs222837 had A-C-C-A/G-C-C-A accumulated sequences.	Balan et al., 2013 [125]
	*Post-mortem* study	Western blot analysis	Human brain tissue (37 SCZ patients, 37 controls)	-	Patients with SCZ had 30% ↓ PSD-95 and 20% ↓ NR1 proteins, so there were fewer functional NMDA receptors in postsynaptic density.	Catts et al., 2015 [126]
	*Post-mortem* study	Western blot analysis	Human brain tissue (SCZ patients: 36ACC + 35DLPFC and controls: 33ACC + 31DLPFC)	-	In ACC, pS295, PSD-95, Rap2, JNK1, and JNK2 were ↓. In the DLPFC, the expression of Cdk5, Rack1, pS295 PSD-95, and NR2B were ↑.	Funk et al., 2012 [127]
	*Post-mortem* study	Western blot analysis, TaqMan PCR	Human brain tissue, mice, rats	HAL (rats)	In SCZ group, there was ↓ PSD-95. The beta variant of PSD-95 was ↑ in the GluN1 KD mouse model of SCZ, while the alpha variant was ↑ in rats HAL-treated.	Funk et al., 2017 [128]
	*Post-mortem* study	TaqMan PCR	Human brain tissue (259 SCZ patients, 188 controls)	-	Six SNPs (rs314253 C/T, rs13331 A/G, rs2242449 C/T, rs390200 A/G, rs507506 A/G, rs739669 A/G) of PSD-95 seemed to not represent a risk factor for SCZ.	Kawashima et al., 2006 [129]
	Association study	Maldi-Tof mass, spectrometry	Human (248 SCZ patients, 208 controls)	-	Two PSD-95 variants (rs2521985 and rs17203281) were not associated with SCZ.	Tsai et al., 2007 [130]
	Association study	PCR, Sanger sequencing	Human (1685 SCZ patients in total, 574 ASD in total, 1793 controls)	-	A carrier of the DLG1-G344R PSD-95 variant was identified in the SCZ group. The DLGAP2-R604C variant was present in ASD group.	Xing et al., 2016 [131]
	*Post-mortem* study	Western blot analysis	Human brain (13 SCZ patients, 8 controls)	-	NR2B and PSD-95 expression in the dorsolateral PFC in the SCZ was ↓.	Kristiansen et al., 2010 [132]
	*Post-mortem* study	In situ Hybridization, Western blotting	Human brain tissue (24 SCZ patients, 16 controls)	-	PSD-95 expression was ↓ in the ACC.	Kristiansen et al., 2006 [133]
	*Post-mortem* study	Immunoprecipitation, TEM, Western blot	Human frontal cortex (15 for each group: SCZ, MDD, BPD, controls)	-	NR2A, PSD-95, CaMKIIα, and CaMKIIβ was ↓ in SCZ. While only NR2A was ↓ in MDD.	Matas et al., 2021 [134]
	*Post-mortem* study	Immunoblotting	Human (20 SCZ patients and 20 controls)	-	Patients in the CA1 region presented ↓ PSD95, synaptophysin, and mGluR1.	Matosin et al., 2016 [111]
	*Post-mortem* study	In situ Hybridization	Human brain tissue (16 SCZ patients, 7 controls)	-	NF-L, NR1, GluR5, and PSD-95 were ↑, while SAP102, PSD-93. and yotiao were ↓ in SCZ.	Mueller et al., 2004 [111]
	Preclinical study	Immunohistochemistry, Western blot	Mice and human brain tissue	-	Synaptic dysbindin-1 was ↓ in the SCZ; synaptophysin or PSD-95 levels did not change.	Talbot et al., 2011 [135]
	*Post-mortem* study	Immunoautoradiography, Western blot	Human brain tissue (15 for each group: SCZ, MDD, BPD, controls)	-	Reduction of PSD-95 at molecular layer glutamate synapses can alter connections to other hippocampal regions.	Toro et al., 2005 [136]
	Preclinical study	In situ hybridization	Rats	HAL, OLA	PSD-95 expression was not changed by administration of typical or atypical AT.	de Bartolomeis et al., 2002 [113]
	Preclinical study	In situ hybridization	Rats	KET, MEM, MK-801	KET and MK-801 ↓PSD-95 in dmCP and dlCP. MEM ↑PSD-95 in dlCP and vmCP.	de Bartolomeis et al., 2013 [114]
	Preclinical study	In situ hybridization	Mice (Ddo^−/−^ and Ddo^+/+^)	PCP	Ddo^−/−^ naive mice present ↓PSD-95 in the striatum and cortex. In M1, mRNA expression of PSD-95 was higher in Ddo^−/−^ PCP mice than in Ddo^−/−^ mice. In addition, gene expression was higher in Ddo^+/+^ PCP mice than in KO mice in both M1 and PrL.	de Bartolomeis et al., 2015 [115]
	Preclinical study	In situ hybridization	Rats	ASE, HAL, OLA	In the cortex, striatum, and NAc, PSD-95 expression was ↑ by both ASE and OLA. While PSD95 was ↑ by HAL in AC, M1, M2, I, dmCP, dlCP, vmCP, NAc.	de Bartolomeis et al., 2015 [107]
	Preclinical study	Autoradiography, In situ hybridization	Rats	CLO, HAL, ZIP	PSD-95 ↑ in the striatum and ACC by administration of HAL, and in the striatum and cortex (ACC, MAC and M1) by ZIP.	Iasevoli et al., 2011 [119]
	Preclinical study	Co-mmunoprecipitation, Western blot analysis	Mice	D-serine	Application of D-serine ↑ SR interactions with PSD-95 and NR1 and ↑ the number of glutamatergic synapses positive at VGLUT1 and PSD-95. D-serine/SR are associated with PSD-95 and NMDAR in postsynaptic neurons and correlate with the stability of glutamatergic synapses during synaptogenesis.	Lin et al., 2016 [137]
	Preclinical study	Electrophysiology, Densitometry, Western blot analysis	Mice (Ns^−/−^)	-	Ns-^/-^ mice showed cognitive deficits and ↓ spinal synapse density in CA1 of the hippocampus, while PSD-95 ↑ in Ns^−/−^ mice. These mice also showed a ↓ LTP.	Reumann et al., 2017 [138]
	In vitro preclinical study	Immunofluorescence, Western blot analysis	Rats	APZ, CLO HAL	APZ and CLO ↑ PSD95 protein levels, the number of spines, phosphorylated Akt Thr308 and Ser473, and phosphorylated GSK-3 beta Ser9. In contrast, HAL or an inappropriate concentration of CLO ↓ them.	Takaki et al., 2018 [139]
	Preclinical study	Immunohistochemistry Transmission electron microscopy assay Western blot analysis	Rats	KET	Ketamine resulted in ↓ BDNF and PSD-95 levels in PCC. Vinpocetine can reverse synaptic ultrastructure by upregulating BDNF-bound PSD-95 reducing ketamine-induced SCZ-like effects in rats.	Xu et al., 2019 [140]
	Preclinical study	PCR Amplification Direct PCR-Sequencing Reaction	Human (523 SCZ patients, 596 controls)	-	Increased expression of the DLGAP2 gene is implicated in the pathogenesis of SCZ.	Li et al., 2014 [141]
SAP97	*-Post-mortem* study -Preclinical study	Western blot analysis	-Human brain (34 SCZ patients, 41 controls) -Rats	HAL (rats)	SAP97 levels is alterated in the PFC of SCZ patients, and this modification of PDZ protein expression is not correlated to AT used.	Toyooka et al., 2002 [142]
SAP102	*Post-mortem* study	In situ Hybridization, Histochemistry	Human brain tissue (60 subjects)	-	In the SCZ, MDD and BD NR1 is ↓. There is a ↓ in NR2A in the SCZ and MDD and a ↓ in NR2C in the SCZ. The expression of SAP102 was ↓ in BD.	Beneyto et al., 2008 [143]
	*Post-mortem* study	Western blotting	Human brain tissue (15 SCZ patients, 8 controls)	-	NR2B and PSD95 are ↑ in the dorsomedial thalamus but not in the ventral thalamus of SCZ patients. While NF-L and SAP102 levels are not different between groups.	Clinton et al., 2006 [144]
	*Post-mortem* study	In situ Hybridization	Human striatum tissue (60 subjects overall, 3 gropus 15 SCZ/ BD /MDD)	-	PSD-95 and SAP-102 in BD and SAP-102 in MDD and SCZ are ↓ in striatum.	Kristansen et al., 2005 [145]
	*Post-mortem* study	In situ Hybridization Western blotting	Human brain tissue (24 SCZ patients, 16 controls	-	A significant ↑ of NR1C20 in the ACC was found in the SCZ patients. Furthermore, the expression of NF-L in DLPFC and PSD-95 and PSD-93 in the ACC are changed. SAP102 expression do not have any modifications.	Kristansien et al., 2006 [133]
	*Post-mortem* study	In situ Hybridization	Human brain (24 subjects overall, 3 gropus 8 SCZ/ BD /MDD)	-	In BD, not in SCZ, ↓ NR1 and NR2A and SAP102 in hippocampus (CA1, CA2, CA3, CA4, DG, and Sub).	McCullmsmith et al., 2007 [146]
	*Post-mortem* study	In situ Hybridization	Human brain (16 SCZ patients, 7 controls)	-	SAP102 was poorly expressed in the SNc	Mueller et al., 2004 [147]
Shank	*Post-mortem* study	Western blotting dot blots	Human brain tissue (20 SCZ patients and 20 controls)	-	Alterations of proteins involved in CME (such as Dynamin-1, adaptor protein 2) and proteins interacting with NMDA were related to SCZ. PSD, NMDA-interacting proteins and endocytosis-related proteins contribute to the pathophysiology of SCZ.	Föcking at al., 2015 [69]
	In vitro preclinical study	Immunocytochemistry, Immunoblotting, Semiconductor sequencing, PCR	Human (98 SCZ and 484 controls)	-	PSD-related genes (such as NLGN, SHANK and DLGAP) had rare functional mutations that could alter protein expression in some patients with SCZ.	Hu et al., 2020 [10]
	Preclinical study	qRT-PCR Immunocytochemistry Western blotting	Human (Patients with 22q13 deletion)	-	PMDS presented ↓ of Shank3 expression in neurons.	Shcheglovitov et al., 2013 [148]
	In vivo preclinical study	In situ hybridization, Histochemistry, Radiolabeling	Rats	Lithium, VPA (in chronic)	Both mood stabilizers ↓ expression of Shank, IP3R, and Homer1b/c correspondingly in cortex and dorsolateral caudate-putamen, whereas only VPA reduced gene expression in all other striatal subregions.	De Bartolomeis et al., 2012 [104]
Spinophilin	*Post-mortem* study	Western blot analysis	Human brain (24 subjects SCZ: 12 AT-free, 12 AT-treated; and 24 controls)	-	Spinophilin lower band (80–95 kDa) showed a significant ↓ in AT-treated dorsolateral PFC of SCZ subjects compared to controls (but not in AT-free).	Brocos et al., 2021 [149]
	*Post-mortem* study	qRT-PCR analysis	Human brain tissue	-	There was a significant negative correlation with interneuronal markers, parvalbumin and somatostatin, while a positive correlation with spinophilin, but not with PSD-95.	Catts et al., 2012 [150]

## Data Availability

The datasets generated and analyzed during the current study are available from the corresponding author on reasonable request.
